# Switchable Bidirectional Sound Absorption Via Exceptional Point Modulation in Acoustic Metastructures with Interleaved Resonator Coupling

**DOI:** 10.1002/advs.202508951

**Published:** 2025-08-11

**Authors:** Zichao Guo, Liangfen Du, Zirui Yang, Kexin Zeng, Ziping Lei, Zhonggang Wang, Zhendong Li, Zheng Fan

**Affiliations:** ^1^ School of Traffic & Transportation Engineering Central South University Changsha Hunan 410075 China; ^2^ School of Mechanical and Aerospace Engineering Nanyang Technological University 50 Nanyang Avenue Singapore 639798 Singapore; ^3^ Department of Building Environment and Energy Engineering The Hong Kong Polytechnic University Hung Hom Kowloon Hong Kong SAR 999077 China; ^4^ Department of Mechanical Engineering National University of Singapore Singapore 117575 Singapore; ^5^ The State Key Laboratory of Heavy‐duty and Express High‐power Electric Locomotive Zhuzhou Hunan 412000 China

**Keywords:** acoustic metastructures, bidirectional absorption, exceptional point, interleaved resonators

## Abstract

Physics‐driven acoustic metamaterials offer unprecedented capabilities in manipulating sound wave propagation. Among these, sound‐absorbing metamaterials emerge as powerful tools for achieving subwavelength control and high‐efficiency absorption. However, most existing designs are typically constrained to unidirectional absorption, limiting their applicability in noise‐sensitive scenarios requiring bidirectional control. Here, a switchable bidirectional acoustic metastructure is presented, integrating interleaved resonator coupling with exceptional point (EP) modulation. By coordinating vertical and horizontal resonant interactions through tailored impedance matching and controlled energy dissipation, the system achieves broadband and frequency‐selective absorption in both directions—validated through theoretical, numerical, and experimental analyses. Specifically, broadband absorption from 478 to 670 Hz and discrete peaks at 260 and 542 Hz under opposite incidences are observed within the deep‐subwavelength scale. Compared to state‐of‐the‐art unidirectional absorbers, the proposed structure maintains geometric compactness while delivering robust bidirectional performance. Beyond device‐level innovation, a generalized theoretical framework is developed to translate the effective acoustic parameters of resonance‐based metastructures into specific impedance forms, enabling integration into EP‐based switching strategies. This enables the functional extension from unidirectional to bidirectional absorption across a wide range of metastructures. Overall, this work offers a novel physics‐driven pathway toward practical, high‐performance, and bidirectional acoustic wave control.

## Graphical abstract

A switchable bidirectional acoustic metastructure is proposed, enabling broadband and frequency‐selective absorption via interleaved resonator coupling and exceptional point modulation. It outperforms traditional unidirectional designs, offering compact, efficient sound control from both directions and establishing a generalized framework for extending EP‐based absorption to diverse local‐resonance sound‐absorbing metamaterials.

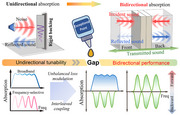

## 1 Introduction

**Figure 1:**
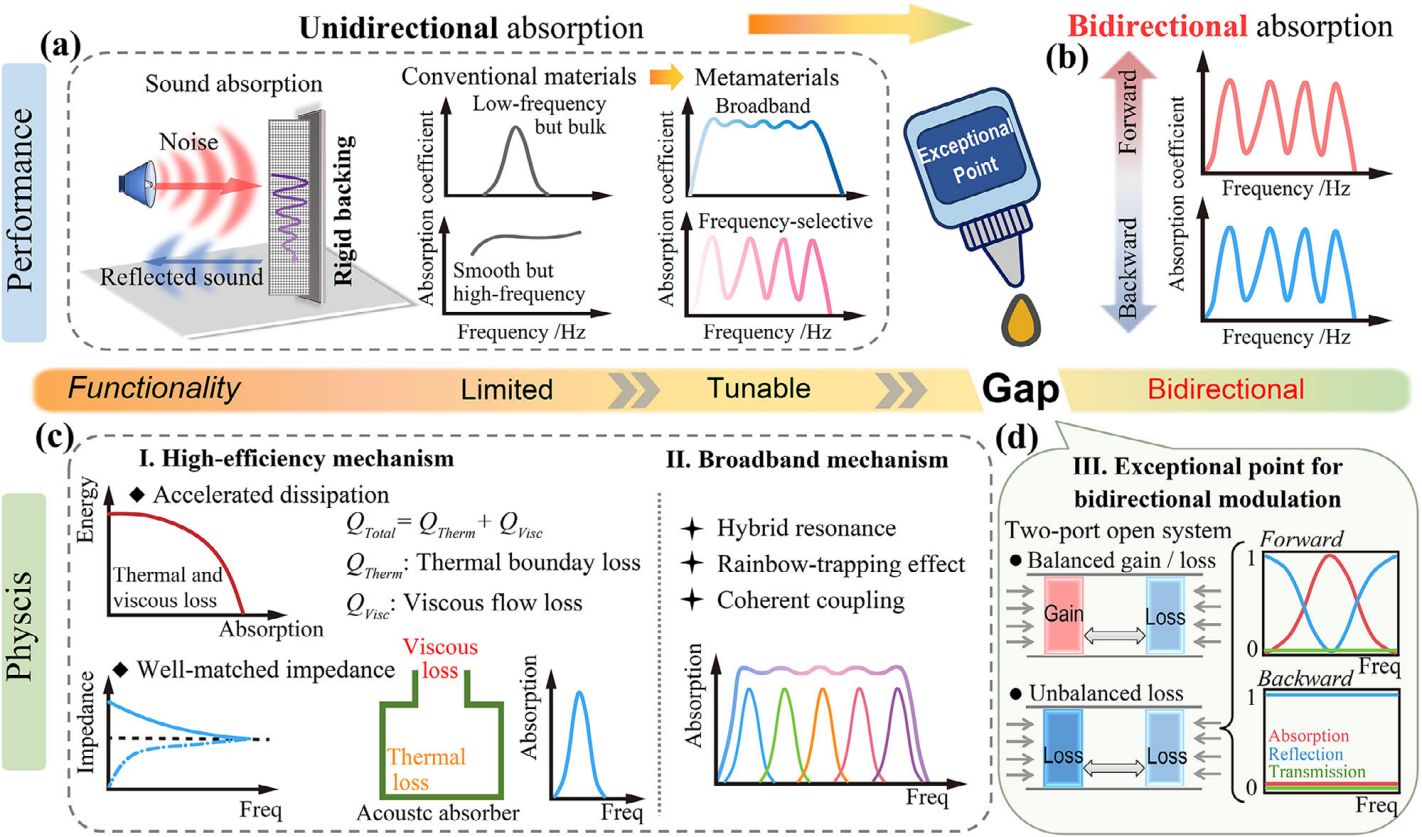
Conceptual framework for bidirectional sound absorption. a) Unidirectional absorption: Conventional materials provide limited sound absorption, while metamaterials enable tunable, broadband, and frequency‐selective absorption. b) Toward bidirectional functionality: EPs enable bidirectional absorption with distinct absorption profiles in forward and backward directions, highlighting a functional gap in current acoustic absorber designs. c) Underlying physical mechanisms of high‐efficiency and broadband absorption: I. Enhanced energy dissipation through viscous and thermal losses; II. Broadband performance enabled by hybrid resonances and coherent coupling. d) EP‐enabled bidirectional modulation strategies: Balanced gain–loss configuration yielding symmetric response; Unbalanced‐loss configuration enabling asymmetric sound control.

Manipulating acoustic wave propagation and energy dissipation presents a fundamental physical challenge with broad implications,^[^ ^1^, ^2^, ^3^, ^4^ ^]^ especially in noise mitigation, vibration isolation, and other wave‐based control applications.^[^ ^5^, ^6^, ^7^ ^]^ In complex acoustic environments, ranging from transportation systems to architectures and industrial facilities, noise not only impairs acoustic comfort but also degrades human health and system performance.^[^ ^8^, ^9^, ^10^ ^]^ The intrinsic constraints of conventional materials, rooted in their atomic‐scale structures and chemical interactions, render them ineffective for attenuating low‐frequency sound due to wavelength‐scale limitations.^[^ ^11^, ^12^, ^13^ ^]^ From a physical standpoint, the inability to dissipate or manipulate the propagation path of acoustic energy at subwavelength scales reveals intrinsic limitations in conventional approaches. These limitations have catalyzed the development of acoustic metamaterials and metastructures that harness local resonances, impedance modulation, and wave–structure coupling to achieve precise control of sound propagation.^[^ ^14^, ^15^, ^16^, ^17^, ^18^ ^]^ By engineering these physical mechanisms, such structures enable rapid attenuation of incident waves and offer broadband, low‐frequency, and frequency‐selective absorption capabilities,^[^ ^19^, ^20^, ^21^, ^22^, ^23^, ^24^ ^]^ as illustrated in **Figure** **1**a,c.

However, most existing designs are limited to unidirectional sound control, offering effective absorption only when sound is incident from a specific direction. As illustrated in Figure 1b, bidirectional sound absorption is essential in many real‐world applications, including open office environments, shared public spaces, and enclosures with sound sources on both sides. Traditional approaches to achieve such bidirectional performance often rely on placing identical absorbers on both sides, resulting in increased system thickness and reduced structural compactness. This highlights the pressing demand for novel mechanisms and structural strategies capable of achieving bidirectional sound absorption within compact and efficient metastructures.

To address this limitation, the concept of exceptional points (EPs) in non‐Hermitian physics has attracted considerable attention for enabling unidirectional response and nonreciprocal wave phenomena.^[^ ^25^, ^26^, ^27^ ^]^ In both classical and quantum physics, non‐Hermitian systems, characterized by complex‐valued effective parameters due to loss, gain, or both, are widely employed to model open systems with intrinsic energy exchange.^[^ ^28^, ^29^, ^30^, ^31^ ^]^ Unlike Hermitian counterparts that conserve energy, these systems inherently exhibit non‐conservative behavior, enabling phenomena such as asymmetric scattering, mode hybridization, and EP degeneracies. These EPs, singularities where two or more eigenvalues and eigenvectors coalesce, have been extensively studied in two‐port open acoustic systems, offering theoretical potential for achieving asymmetric acoustic responses. In the exploration of non‐Hermitian acoustics, the emergence and interaction of EPs can be realized through two main approaches: introducing asymmetric loss distributions connected to acoustic cavities,^[^ ^32^, ^33^, ^34^ ^]^ or employing gain–loss‐balanced media such as Parity‐Time symmetric systems.^[^ ^35^, ^36^, ^37^ ^]^ EP‐induced systems are promising platforms for asymmetric sound absorbers due to their inherent unidirectional and asymmetric wave propagation characteristics,^[^ ^32^, ^34^ ^]^ as shown in Figure 1d. Most existing studies focus on achieving extreme asymmetry, aiming for near‐perfect absorption from one side and near‐zero from the other. However, the physical mechanisms that enable tunable bidirectional absorption remain poorly understood. These include coupling‐state modulation, energy dissipation, EP modulation, and impedance matching. Clarifying how these mechanisms interact is crucial for achieving robust and tunable bidirectional sound absorption.

Motivated by this gap, this work introduces a switchable directional acoustic absorber that integrates the bidirectional manipulation of EP with a structure‐responsive transformation strategy. By leveraging interleaved resonator coupling along both vertical and horizontal dimensions, the proposed metastructure enables bidirectional control of acoustic waves across a wide frequency range. It achieves frequency‐selective or broadband sound absorption across a wide operating band, with experimental, theoretical, and numerical validations confirming near‐perfect absorption from both directions. Specifically, broadband absorption from 478 to 670 Hz was observed under forward incidence, along with discrete peaks at 260 and 542 Hz under backward incidence, all exhibiting deep‐subwavelength behavior. Compared to state‐of‐the‐art acoustic metastructures, the proposed design not only achieves balanced, high‐efficiency low‐frequency and broadband performance, but also maintains bidirectional response robustness without compromising compactness.

Furthermore, this work not only demonstrates novel bidirectional absorption capability but also establishes a generalized theoretical framework. By extending the EP‐based impedance modulation strategy, the framework enables broader applicability across a wide range of resonance‐based or porous acoustic metastructures. This offers a new pathway to transition their absorption performance from unidirectional to bidirectional operation, thereby enhancing both their physical capabilities and engineering adaptability. Overall, this work presents a switchable bidirectional acoustic absorption strategy via interleaved resonator coupling and EP modulation, demonstrating strong potential in terms of performance, tunability, practical implementation, and theoretical generality.

Beyond its contribution to wave control, this physics‐driven strategy offers valuable insights for the development of multifunctional acoustic devices. Such devices aim to integrate sound absorption with additional functionalities, as demonstrated in recent studies.^[^ ^38^, ^39^, ^40^ ^]^ By providing a tunable and scalable platform, the present work opens promising avenues for future integration into multifunctional metastructures for advanced noise‐control applications.

## 2 Interleaved Resonator Coupling Strategy

### 2.1 Accessing Exceptional Point Modulation

**Figure 2:**
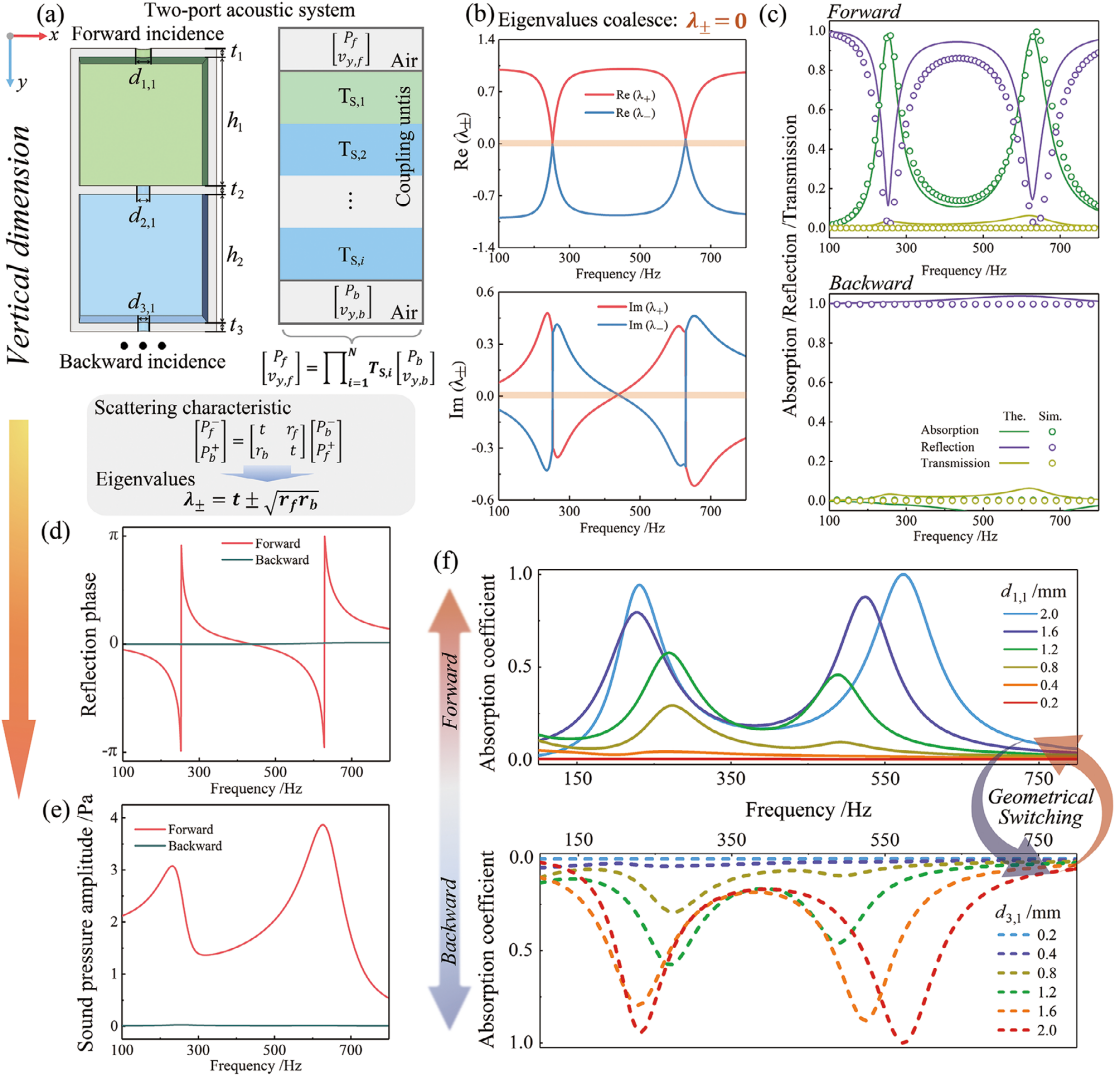
Asymmetric sound absorption enabled by EP‐based mechanisms via vertical dimension modulation. a) Prototypes of asymmetric absorbers featuring a cascade coupling design. b) Real and imaginary parts of the eigenvalues λ_+_ and λ_−_ across the operating frequency range. c) Absorption, reflection, and transmission coefficients under forward and backward incidence, solid and dashed lines represent results from theoretical modeling and numerical simulations, respectively. d) Theoretically calculated reflection phase responses for forward and backward incidences. e) Sound pressure amplitude spectra under forward and backward excitation. f) Absorption spectra under varying pore diameters *d*_1,1_ and *d*_3,1_; curves with identical color represent the same geometric configuration, illustrating direction‐dependent absorption performance.

Acoustic resonances are a universal phenomenon in nature, particularly prominent in open systems where interaction and energy exchange with the surrounding environment lead to complex eigenfrequencies. Such systems are inherently non‐Hermitian, as they deviate from ideal energy conservation due to the presence of loss, gain, or both. Helmholtz resonators (HRs) serve as classical examples of this behavior.^[^ ^41^, ^42^ ^]^ In this context, the two‐port acoustic system proposed in this study, as illustrated in **Figure** **2**a, is fundamentally non‐Hermitian. This classification arises from the presence of energy dissipation mechanisms, specifically viscous and thermal losses within the micro‐perforated plates (MPPs) and connected cavities. These losses introduce complex‐valued acoustic parameters, resulting in non‐conservative wave propagation and characteristic features of open, lossy systems. To theoretically analyze the acoustical performance of this lossy non‐Hermitian system, a standard two‐port model is employed to describe the acoustic response of asymmetric absorbers, expressed as

(1) $$\left[\begin{array}{c} P_{f}\\ v_{y,f} \end{array}\right]=T_{S,total}\;\left[\begin{array}{c} P_{b}\\ v_{y,b} \end{array}\right]$$

where *P_f_* and *P_b_* represent the forward and backward complex pressure amplitudes at the two terminals of this cascade structure, *v*_*y*,*f*_ and *v*_*y*,*b*_ are the associated forward and backward velocity fields. Here, the term “forward” refers to sound waves entering from the top side of the structure, while “backward” corresponds to entry from the bottom side. Further, the total transfer matrix *T*_S,*total*_ with *N*‐layer HRs is expressed as

(2) $$T_{S,total}=\prod_{i=1}^{N}T_{S,i}=\left[\begin{array}{cc} T_{11} & T_{12}\\ T_{21} & T_{22} \end{array}\right]=T_{p1}\;T_{c1}T_{p2}T_{c2}T_{p3}\text{…}$$

where *T_p_* describes the matrices of the plates, respectively; *T_c_* represents the matrix of the cavity, calculated as

(3) $$T_{p}=\left[\begin{array}{cc} 1 & Z_{p}\\ 0 & 1 \end{array}\right]$$

(4) $$T_{c}=\left[\begin{array}{cc} \cos\left(kh_{i-1}\right) & jZ_{0}\sin\left(kh_{i-1}\right)\\ j\sin\left(kh_{i-1}\right)/Z_{0} & \cos\left(kh_{i-1}\right)\; \end{array}\right]$$

where $Z_{0}=\sqrt{\rho_{0}/K_{0}^{-1}}$ is the characteristic impedance of the background medium. *Z_p_* denotes the impedance of MPP and is expressed as^[^ ^43^ ^]^

(5) $$\begin{array}{ccc} Z_{MPP} & = & \frac{j\omega \rho_{0}t_{i}}{\sigma_{i,1}}\;{\left[1-\frac{2B_{1}\left(\eta \sqrt{-j}\right)}{\left(\eta \sqrt{-j}\right)B_{0}\left(\eta \sqrt{-j}\right)}\right]}^{-1}\\ & & +\frac{\sqrt{2}\mu \eta }{\sigma_{i,1}d_{i,1}}+\frac{0.85j\omega \rho_{0}d_{i,1}}{\sigma_{i,1}} \end{array}$$

where $\eta =d_{i,1}\;\sqrt{\omega \rho_{0}/4\mu }$ represents the ratio of the diameter of the perforation to the thickness of the viscous boundary layer. For clarity, the key physical and geometric parameters used are summarized in **Table** **1**. Hence, the corresponding scattering coefficients of the asymmetric absorber can be given as:^[^ ^44^ ^]^

(6) $$t=\frac{2e^{jkL}}{T_{11}+T_{12}/Z_{0}+T_{21}Z_{0}+T_{22}}$$

(7) $$r_{f}=\frac{T_{11}+T_{12}/Z_{0}-T_{21}Z_{0}-T_{22}}{T_{11}+T_{12}/Z_{0}+T_{21}Z_{0}+T_{22}}$$

(8) $$r_{b}=\frac{-T_{11}+T_{12}/Z_{0}-T_{21}Z_{0}+T_{22}}{T_{11}+T_{12}/Z_{0}+T_{21}Z_{0}+T_{22}}$$

where *t* is the transmission coefficient; *r_f_* and *r_b_* denote the reflection coefficients for sound radiated from the forward and backward sides, respectively. The asymmetric absorption performance from different ports can be derived as

(9) $$\alpha_{f}=1-{\left|r_{f}\right|}^{2}-{\left|t\right|}^{2}$$

(10) $$\alpha_{b}=1-{\left|r_{b}\right|}^{2}-{\left|t\right|}^{2}$$

**Table 1:** Definitions of geometric and physical parameters used in analytical modeling.

Parameter	Definition
*d_i_*	Diameters of perforated pores in the *i*‐th unit.
*t_i_*	Thickness of perforated pores in the *i*‐th unit.
*h_i_*	Height of the connected cavity behind the 𝑖‐th perforated plate.
*L*	Total thickness of the entire absorber structure.
σ_*i*_	Perforation ratio of the 𝑖‐th plate.
*k*	Acoustic wavenumber.
ρ_0_	Density of air (typically 1.21 kg m^−^^3^ under standard conditions)
*K*_0_	Bulk modulus of air
μ	Dynamic viscosity (1.8 × 10^−5^ Pa · s)
*B*_0_	Zeroth order Bessel functions
*B*_1_	First order Bessel functions

Furthermore, the scattering characteristic of this asymmetric two‐port system is described by its scattering matrix. The forward and backward ingoing acoustic waves are denoted by $P_{f}^{+}$ and $P_{b}^{-}$, while the outgoing sound waves are expressed as $P_{f}^{-}$ and $P_{b}^{+}$, respectively. The relation is expressed by

(11) $$\left[\begin{array}{c} P_{f}^{-}\\ P_{b}^{+} \end{array}\right]=\left[\begin{array}{cc} t & r_{f}\\ r_{b} & t \end{array}\right]\;\left[\begin{array}{c} P_{b}^{-}\\ P_{f}^{+} \end{array}\right]$$

The eigenvalues of the scattering characteristic can be described as a 2 × 2 matrix, given by

(12) $$\lambda_{\pm }=t\pm \sqrt{r_{f}r_{b}}$$

Building upon the prior equations, the system exhibits asymmetric scattering behavior, where the reflection coefficients under forward and backward incidence are generally unequal. The evolution of eigenvalues λ_±_ is dictated by the tuning of asymmetric losses, which are physically induced by variations in geometric parameters.

Notably, under specific geometrical and loss‐tuning conditions in the non‐Hermitian system, the two eigenvalues or eigenstates coalesce, signifying the presence of an exceptional point (EP), as illustrated in Figure 2b. The EP is characterized by the coalescence of both the real and imaginary parts of the eigenvalues, leading to highly asymmetric wave responses under forward and backward incidence. Accordingly, the scattering coefficients for both forward and backward incidence are shown in Figure 2c, validated through theoretical analysis and numerical simulations. The numerical simulation method is illustrated in Section S1 (Supporting Information). When acoustic waves impinge from the forward direction, they are almost completely or entirely absorbed, resulting in near‐perfect or perfect absorption. In contrast, backward incidence leads to near‐total reflection with negligible absorption, demonstrating asymmetric acoustic absorption. Moreover, due to the cascade configuration of two or more HRs, near‐perfect or perfect absorption is achieved at their respective resonance frequencies.

Furthermore, Figure 2d presents the reflection phase responses under forward and backward incidence. The asymmetric system undergoes an abrupt reflection phase transition when excited from the forward direction. At this point, the reflection vanishes due to the emergence of EPs, thereby enabling unidirectional reflection. In addition, Figure 2e shows that, at resonance frequencies associated with the EP regime, the local sound pressure amplitudes within the operating HRs are substantially enhanced. This confirms strong energy localization and dissipation. The corresponding sound pressure field distributions are provided in Section S2 (Supporting Information). These results demonstrate that vertical dimensional tuning provides an effective route to manipulate eigenvalue degeneracy and access EPs in asymmetric acoustic systems.

Figure 2f shows the forward and backward absorption spectra of the system under variation of the loss‐related geometric parameters *d*_1,1_ and *d*_3,1_, which significantly influence acoustic dissipation. Solid lines correspond to forward incidence, while dashed lines of the same color represent the backward absorption, enabling a direct comparison of the two directions for the same acoustic system. Through the modulation of *d*_1,1_and *d*_3,1_, the system demonstrates two distinct characteristics: a transition of extreme asymmetric absorption direction and efficient bidirectional absorption. The observed spectra highlight the critical role of geometric switching in modulating energy dissipation and achieving switchable sound control. Notably, the internal hole *d*_2,1_ serves as an additional loss factor linking the upper and lower cavities, thereby facilitating cascaded resonance and supporting asymmetric wave responses essential for EP modulation. Further details are provided in Section S3 (Supporting Information). This vertical modulation strategy thus enables not only highly asymmetric responses, but also tunable bidirectional absorption, highlighting its potential for wave control in non‐Hermitian acoustic metastructures.

### 2.2 Tailoring Coherent Coupling Interactions

**Figure 3:**
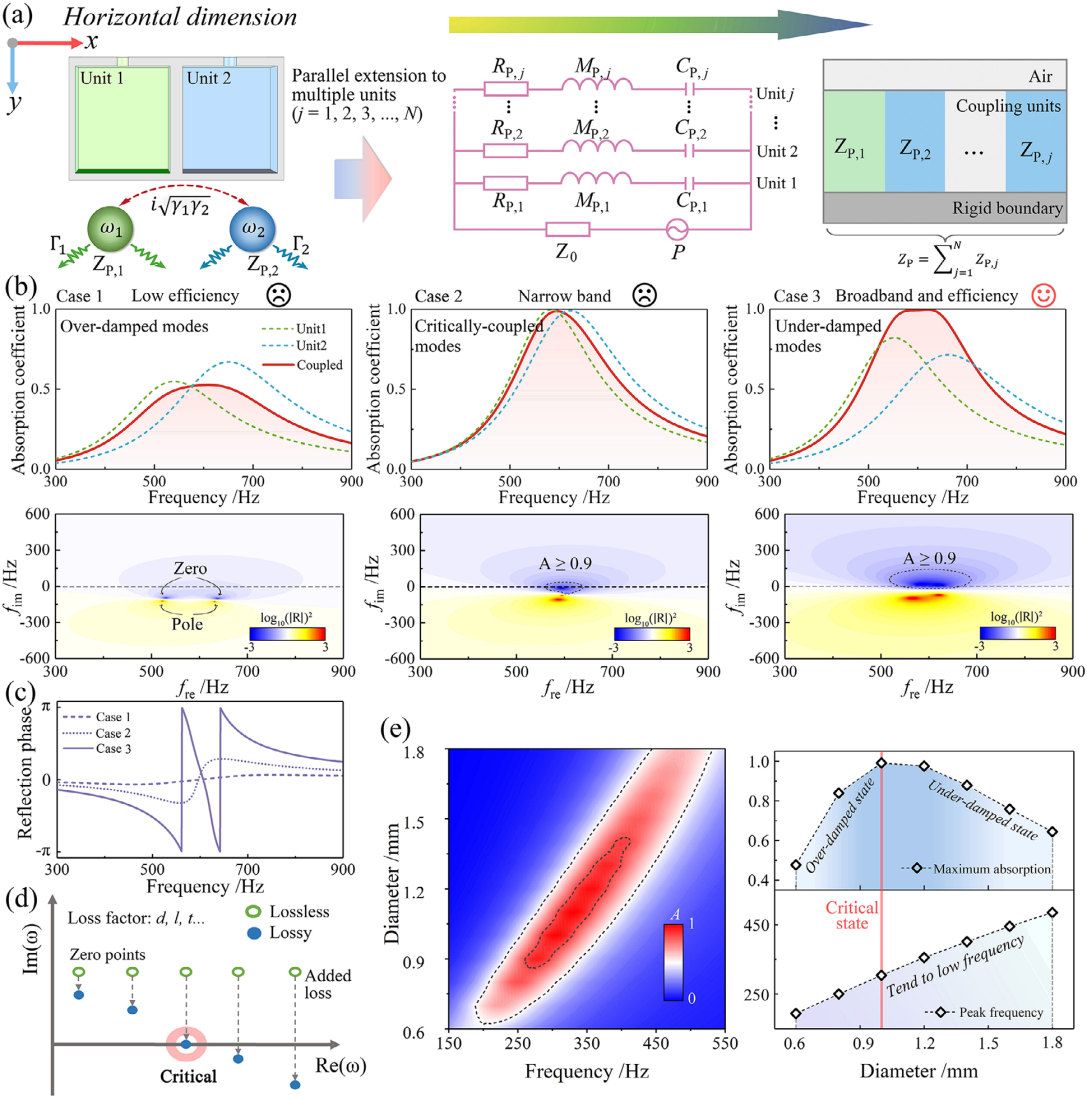
Sound absorption behavior enabled by coherent coupling via horizontal dimensional modulation. a) Schematic of parallel HRs illustrating multi‐coupling behavior. The configuration is extensible to multiple units, with the equivalent circuit representing their impedance relationship. b) Absorption spectra for three representative cases: over‐damped modes, critically‐coupled modes, and under‐damped modes, along with their corresponding reflection coefficients in the complex frequency plane. c) Reflection phase spectra for the three coupling cases. d) Reflection zeros in the complex frequency plane, showing the transition of an HR from lossless (hollow circles) to lossy (filled circles) states by tuning the loss‐related parameters. e) Absorption spectra under varying pore diameters, showing the peak absorption coefficients and corresponding resonant frequency shifts.

The former design achieves asymmetric absorption performance under forward and backward incidence. However, only one or a few discrete absorption peaks exist with this vertical dimensional configuration. This limitation hinders its applicability in scenarios requiring broadband or continuous frequency‐selective acoustic control. To overcome this issue, coherent coupling between parallel resonators arranged along the horizontal dimension is introduced, enabling strong coupling interactions and facilitating broadband, high‐efficiency absorption. Here, two HRs are utilized as an example to illustrate the parallel performance, as shown in **Figure** **3**a. Owing to the intrinsic energy dissipation of the resonator and the leakage of acoustic energy into the surrounding medium, the resonance equation with loss is expressed as

(13) $$\frac{d^{2}\psi }{dt^{2}}+\omega_{j}Q^{-1}\frac{d\psi }{dt}+\omega_{j}^{2}\psi =0$$

where ψ represents the time‐dependent resonance function and ω_*j*_ denotes the angular frequency at resonance. *Q* ^−1^ refers to the quality factor, describing the total dissipation of the system, which is comprised of the loss factor $Q_{loss}^{-1}$ and leakage factor $Q_{leak}^{-1}$ ($Q_{loss}^{-1}=2\Gamma /\omega_{j}$ and $Q_{leak}^{-1}=2\gamma /\omega_{j}$, with γ and Γ representing the radiative and dissipative decay rates). To describe the system's resonance behavior, we employ coupled‐mode theory (CMT), which models the interaction between resonant modes and external wave excitation via modal amplitude dynamics.^[^ ^45^, ^46^, ^47^ ^]^ This framework allows analytical expressions for reflection, transmission, and absorption to be derived based on the balance between internal loss and external leakage. Under this theory, the reflection coefficient for a one‐port system is:

(14) $$r=1-\frac{2Q_{leak}^{-1}}{2i\left(\frac{\omega }{\omega_{j}}-1\right)+Q_{leak}^{-1}+Q_{loss}^{-1}}$$

Perfect absorption can be obtained by the “critical coupling” condition, met by Γ  =  γ at the resonance frequency. This energy‐based coupling behavior not only manifests in mode interactions but can also be described via impedance modeling. For multi‐resonator configurations arranged in parallel, their acoustic response can be equivalently analyzed using an acoustic‐electrical analogy, where each resonator, indexed by *j*, is treated as an individual impedance element. The total surface impedance for the parallel configuration is then given by

(15) $$Z_{P}=\sum_{j=1}^{N}\delta_{j}Z_{P,j}$$

where *Z*_P,*j*_ and δ_*j*_ refer to the surface impedance of the corresponding sound‐absorbing resonator and the area ratio of each unit with the parallel absorbers, respectively. It should be noted that Equation (15) is most accurate in the subwavelength regime, where unit dimensions are much smaller than the wavelength.^[^ ^43^, ^48^ ^]^ In this case, radiation effects and mutual coupling are negligible, allowing the surface impedance to be approximated as a weighted sum of individual elements. At higher frequencies, however, radiation impedance and inter‐resonator coupling may introduce deviations. In this work, the subwavelength condition is maintained across the operating range, supporting the validity of the simplified model. The high‐efficiency broadband absorption of the system is modulated via approaching the critical coupling condition. Figure 3b presents the sound absorption spectra in the frequency domain under three different damping states involving two lossy absorbers. The corresponding reflection coefficients are plotted in the complex frequency plane, demonstrating the mode coupling behavior under varying loss conditions. These dissipation behaviors fall into three typical damping regimes. Perfect absorption occurs only under the critical coupling condition when these two factors are matched. A visual comparison and further discussion are provided in Section S4 (Supporting Information). In the first case, both units are over‐damped, leading to weak absorption due to excessive dissipative losses and a suppressed coupling response. In the second case, near‐perfect absorption is achieved by coupling two critically damped units. However, the absorption bandwidth remains narrow, as each unit operates precisely at its critical damping condition, thereby limiting the overall frequency coverage. In contrast, the third case employs two under‐damped resonators and yields a broadband, high‐efficiency absorption response, highlighting the advantage of controlled under‐damping. From a physical perspective, the modulation of each unit aims to balance the intrinsic losses and external leakage of the coupled system. For example, the over‐damped state results in weak acoustic responses, often manifesting as narrowband or inefficient absorption. This behavior corresponds to the presence of zeros in the lower half of the complex frequency plane, which mathematically satisfies the condition Γ > γ. Furthermore, Figure 3c shows that the reflection phase responses of the three cases exhibit distinct features, reflecting the different damping conditions of the system. The reflection phase in Cases 1 and 2 changes gradually or shows slight fluctuations, indicating a non‐ideal coupling state. In contrast, Case 3 shows sharp phase jumps near ± π at two resonant frequencies, revealing a critical state with strong mode coupling and efficient energy trapping. This leads to broadband, high‐efficiency absorption across a wider frequency range.

To further elucidate the role of loss modulation, Figure 3d illustrates the distribution of reflection zeros in the complex frequency plane for a one‐port acoustic system under variations in representative loss parameters (e.g., pore diameter *d*, cavity length *l*, and plate thickness *t*). In the lossless case (green), zeros lie in the upper half‐plane and do not contribute to absorption. When loss is introduced (blue), the zeros shift downward toward the real axis due to increased dissipation. Perfect absorption occurs when a zero lands on the real axis, marking the critical coupling condition in which radiative and dissipative loss rates are balanced. A further increase in loss pushes the zero deeper into the lower half‐plane, resulting in over‐damped coupling and diminished absorption performance. Here, the pore diameter is selected as a representative loss factor to illustrate the transition between under‐damped and over‐damped regimes, as shown in Figure 3e. Additional analysis of other geometric parameters is provided in Section S5 (Supporting Information). In the left panel, a continuous red region indicates high absorption, with the peak shifting toward lower frequencies as the pore diameter increases. According to the principles of Helmholtz resonance, increasing the pore size facilitates greater airflow. This reduces the effective acoustic mass and stiffness of the vibrating air column. Consequently, the system exhibits acoustic impedance mismatch, leading to a reduction in absorption intensity. The right panel further reveals that maximum absorption reaches unity (perfect absorption) at a critical diameter, which separates the under‐damped and over‐damped regimes. Meanwhile, the peak absorption frequency decreases monotonically, confirming that geometric tuning modulates both resonance and damping characteristics. This behavior highlights a viable strategy for achieving critical coupling in the broadband domain through structural parameter optimization.

## 3 Functionality Limitation Breakthrough

Achieving robust and tunable bidirectional sound absorption has long been a challenge in acoustic metastructure design, primarily due to the lack of a clear understanding of the physical mechanisms governing bidirectional control of sound attenuation. This work addresses the limitation by enabling a transition from conventional unidirectional absorbers to switchable bidirectional sound‐absorbing metastructures, realized through the integration of interleaved resonator coupling and EP modulation within a hierarchical recursive design. By leveraging these strategies, the system achieves robust bidirectional absorption while maintaining geometric compactness and high efficiency. The following sections detail the hierarchical design, switching mechanisms, and functional absorption performance of the proposed metastructures.

### 3.1 Hierarchical Recursive Subdivision Design

**Figure 4:**
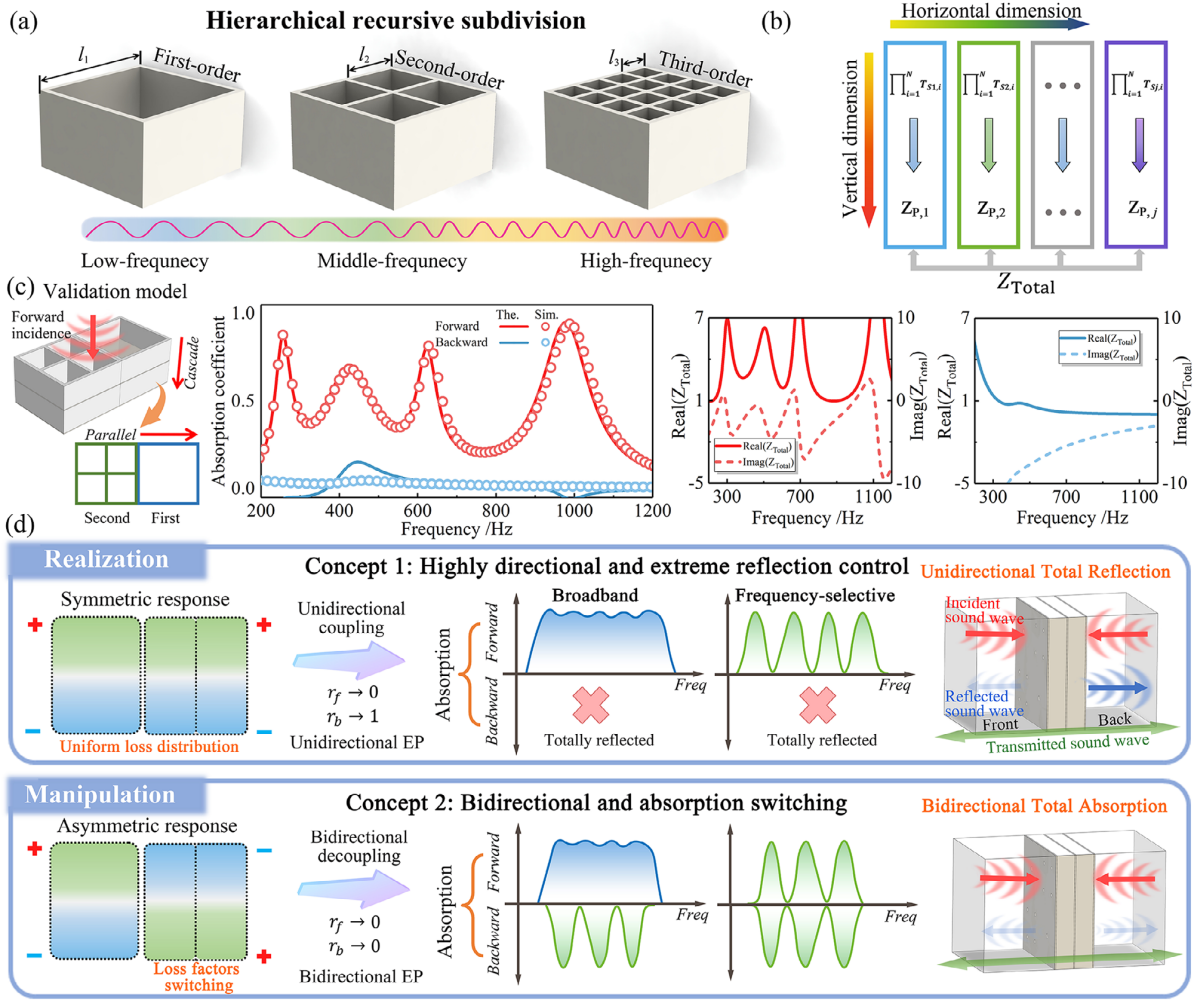
Design strategy and physical mechanisms of direction‐switchable acoustic absorption. a) Hierarchical recursive subdivision design enabling multiscale cavity generation for multiband sound absorption. Subdivided units of increasing order target low‐, mid‐, and high‐frequency ranges. b) Impedance analysis framework for interleaved resonator arrays arranged in cascade and parallel configurations along vertical and horizontal dimensions. c) Validation model with theoretical and numerical comparison of absorption spectra and corresponding total impedance results under forward and backward incidences. d) Two physical concepts for directional absorption manipulation. Concept 1: Unidirectional coupling induced by symmetric response. Concept 2: Loss factors switching introduces asymmetric response and bidirectional decoupling.

**Figure** **4**a illustrates a hierarchical recursive subdivision strategy for broadband acoustic absorption. The configuration starts from a single macro‐scale cavity, which is recursively subdivided into multiple smaller sub‐cavities. At each stage, the existing unit is divided into a 2 × 2 array of equal‐sized elements, resulting in increasingly complex structures with reduced feature sizes from left to right. These configurations are referred to as first‐order, second‐order, and third‐order structures, respectively. This classification is inspired by self‐similar hierarchical designs commonly used in metamaterials,^[^ ^42^ ^]^ and introduces multiple resonant length scales into the structure. The characteristic side length of each sub‐cavity is denoted as *l_x_*, where *x* indicates the hierarchical order. This hierarchical design enables resonance responses across a wide frequency range: 1) the large‐scale cavity supports low‐frequency modes; 2) intermediate subdivisions activate mid‐frequency resonances; and 3) the smallest‐scale units contribute to high‐frequency behavior. This design principle facilitates both broadband and frequency‐selective sound absorption by integrating multiple resonant mechanisms across hierarchical levels.^[^ ^19^ ^]^ It provides a scalable and efficient route to tailor the absorption spectrum through geometric modulation alone, making it particularly advantageous for low‐frequency noise control in engineering applications.

When coupling two‐port cascaded components of different scales in parallel, the overall acoustic system exhibits multi‐scale interleaved impedance characteristics, as illustrated in Figure 4b. For mathematical convenience, the transfer matrices of each individual component are first converted into their corresponding admittance matrices (*Y^j^*) before being combined^[^ ^49^ ^]^

(16) $$Y^{j}=\left[\begin{array}{cc} Y_{11}^{j} & Y_{12}^{j}\\ Y_{21}^{j} & Y_{22}^{j} \end{array}\right]=\frac{1}{T_{12}^{j}}\;\left[\begin{array}{cc} T_{22}^{j} & T_{12}^{j}T_{21}^{j}-T_{11}^{j}T_{22}^{j}\\ 1 & -T_{11}^{j} \end{array}\right]$$

Once the admittance matrices of the individual components are derived, the overall transfer matrix is developed to characterize the acoustic response of the cascade‐parallel absorber. This matrix, denoted as $T_{S,P}^{t}$, can be calculated through the following transformation:

(17) $$\begin{array}{cc} T_{S,P}^{t} & =\left[\begin{array}{cc} T_{11}^{t} & T_{12}^{t}\\ T_{21}^{t} & T_{22}^{t} \end{array}\right]\\ & =\frac{-1}{\sum \delta_{j}Y_{21}^{j}}\;\left(\begin{array}{cc} \sum \delta_{j}Y_{21}^{j} & -1\\ \sum \delta_{j}Y_{22}^{j}\sum \delta_{j}Y_{11}^{j}-\sum \delta_{j}Y_{12}^{j}\sum \delta_{j}Y_{21}^{j} & -\sum \delta_{j}Y_{11}^{j} \end{array}\right) \end{array}$$

Specifically, the total transfer matrix $T_{S,P}^{t}$ models the hybrid resonator system, where each unit's contribution is governed by its surface acoustic impedance and cross‐sectional ratio δ_*j*_. This formulation enables the coupling of multiple resonators into a unified model, allowing the two‐port relation $\left[\begin{array}{c} P_{f}\\ v_{f} \end{array}\right]=T_{S,P}^{t}\;\left[\begin{array}{c} P_{b}\\ v_{b} \end{array}\right]$, to be applied, as introduced in Equation (1). By further applying Equations (6)–(8), the scattering parameters, including reflection and transmission coefficients, can be calculated. This establishes a direct and physically intuitive link between the hybrid geometry of the parallel configuration and its absorption behavior, enabling efficient prediction and design of broadband or direction‐dependent acoustic performance. A validation model is used to verify the consistency between numerical and theoretical results, as shown in Figure 4c. The prototypical metastructure consists of first‐ and second‐order square components arranged in two parallel layers. The corresponding geometric parameters are provided in Section S6 (Supporting Information). The numerical and theoretical results show good agreement, exhibiting asymmetric absorption performance. Meanwhile, the corresponding acoustic impedance matching conditions align well with the observed absorption spectra. Under forward side incidence, well‐matched impedance is observed at the resonance peaks, indicating efficient energy dissipation. In contrast, backward incidence leads to significant mismatches in both the real and imaginary parts of the impedance, deviating substantially from the impedance matching condition.

### 3.2 Switching Mechanism for Bidirectional Acoustic Absorption

The interleaved extension strategy offers precise tunability in both vertical and horizontal dimensions, enabling fine control over the impedance matching characteristics of each component. As a result, the resonant system can be reconfigured to operate in either broadband or frequency‐selective sound absorption modes.

Figure 4d schematically illustrates two physical concepts for tailoring acoustic wave propagation through coupling interaction and geometric symmetry control. In Concept 1, the structure adopts a geometrically symmetric configuration that yields identical responses under both incidence directions. This arrangement induces unidirectional coupling between adjacent resonators, attributed to non‐Hermitian eigenmodes excited by identically oriented loss factor configurations. Under forward incidence, these directionally induced modes are coherently excited, facilitating impedance matching and strong mode hybridization. This interaction leads to efficient energy dissipation, which manifests as either broadband or frequency‐selective sound absorption. Conversely, backward incidence fails to excite the same coupled modes, leading to impedance mismatch and minimal energy coupling, and hence near‐total reflection. This direction‐dependent behavior arises from the asymmetric excitation of coupling modes and corresponds to the system operating near a unidirectional EP. Physically, it reflects the coalescence of eigenmodes and eigenvalues in only one propagation direction, enabling unidirectional total reflection ($r_{f}\to 0,r_{b}\to 1$), which is analogous to a diode‐like behavior.

Building on this directional mechanism, Concept 2 adopts a loss‐factor switching strategy that eliminates unidirectional dependence and enables bidirectional absorption by independently tuning asymmetric coupling in each direction. This loss‐factor inversion redistributes the resonance mode profiles and establishes effective decoupling between ports, resulting in a balanced interaction of forward and backward propagating waves. Decoupling the mode interactions at each port releases direction‐specific constraints, enabling the system to achieve consistent and tunable absorption under bidirectional excitation. Both incidence directions independently satisfy the critical coupling condition via well‐matched impedance, ensuring minimal reflection and maximal energy dissipation ($r_{f}\to 0,r_{b}\to 0$). The system thus operates under directionally engineered loss distributions, effectively establishing a loss‐controlled bidirectional EP regime that enables symmetric and high‐efficiency sound absorption.

### 3.3 Functional Absorption Performance

**Figure 5:**
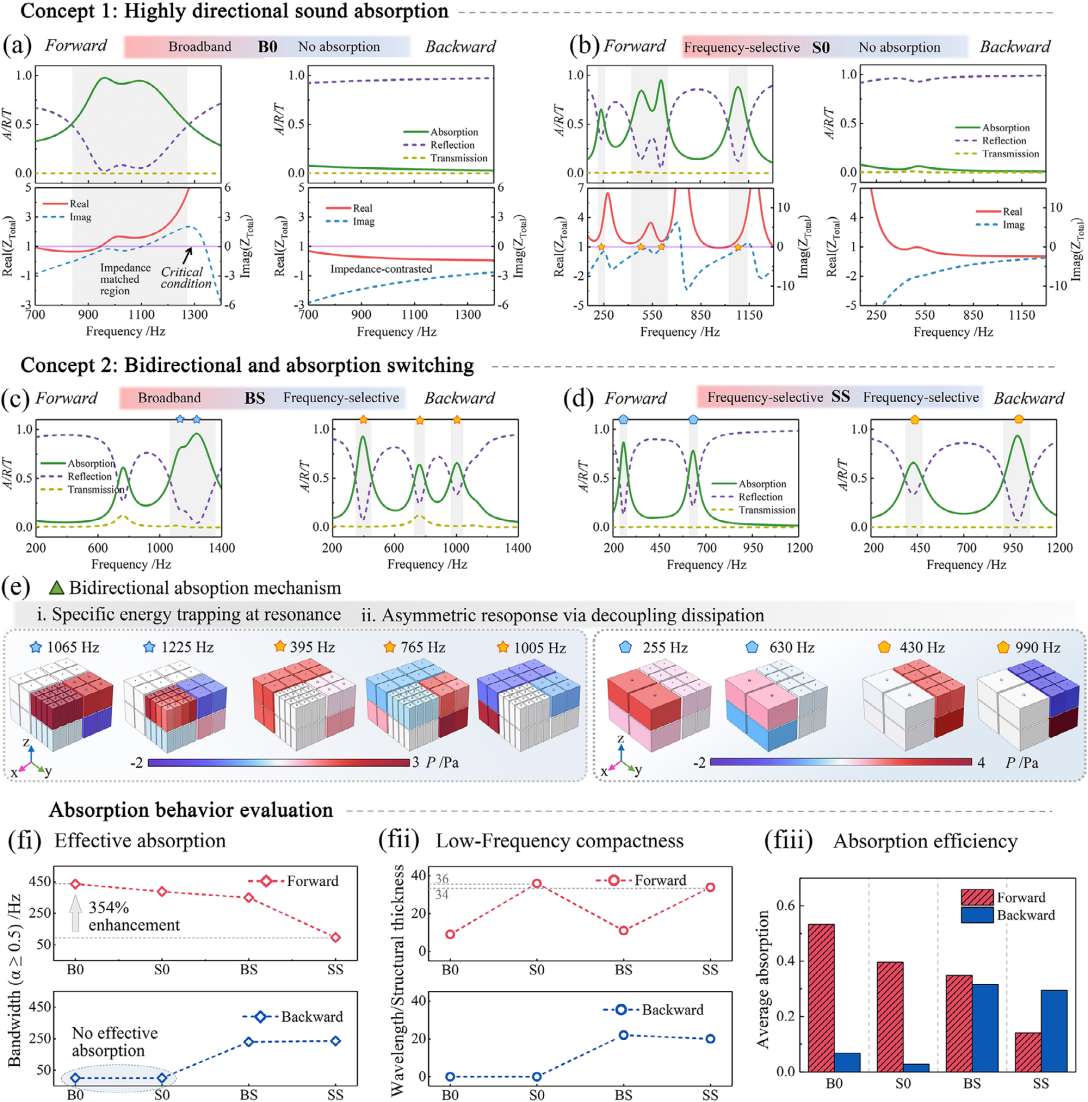
Unidirectional and bidirectional sound absorption performance. a,b) Concept 1: Unidirectional sound absorption. The forward incidence exhibits strong broadband or frequency‐selective absorption with well‐matched impedance, whereas the backward incidence results in total reflection due to severe impedance mismatch. c–e) Concept 2: Bidirectional and switchable absorption. Critical coupling conditions are satisfied in both directions, enabling broadband or frequency‐selective absorption responses. e) Physical mechanisms of bidirectional absorption illustrated by sound pressure distributions at representative resonance frequencies. (f) Quantitative evaluation of absorption behavior, including f‐i) effective absorption bandwidth (α ≥ 0.5), f‐ii) low‐frequency subwavelength performance, and f‐iii) average absorption within the 200–1400 Hz range.

Based on the preceding analysis, two fundamental design strategies emerge for directionally actuated functional sound absorption: highly directional absorption and switchable bidirectional absorption. These two mechanisms are systematically analyzed in **Figure** **5**, along with their underlying physical principles. For clarity, a set of abbreviations is introduced to represent absorption behavior under forward and backward incidence: B denotes broadband absorption, S indicates frequency‐selective absorption, and 0 corresponds to negligible (zero) absorption. For example, BS denotes broadband absorption in one direction and frequency‐selective absorption in the opposite direction; S0 corresponds to frequency‐selective absorption in one direction and negligible absorption in the reverse. The four performance configurations: B0, S0, BS, and SS are categorized into the two conceptual strategies mentioned above, as summarized in **Table** **2**. The corresponding geometric parameters of each hierarchical component are provided in Section S6 (Supporting Information).

**Table 2:** Definition of absorption behavior labels for bidirectional acoustic performance.

Label	Forward incidence	Backward incidence
B0	Broadband	Zero
S0	Frequency‐selective	Zero
BS	Broadband	Frequency‐selective
SS	Frequency‐selective	Frequency‐selective

In Concept 1, the B0 and S0 configurations, constructed from two layers of hierarchical components with a total thickness of 40 mm, exhibit strongly directional absorption behavior, as shown in Figure 5a,b. From forward incidence, the coupled absorbers produce either broadband or frequency‐selective sound absorption, associated with clear impedance matching at resonances—evident from the alignment of real and imaginary parts of the total impedance (*Z_Total_*). These responses correspond to a near‐critical coupling regime, where both the energy dissipation efficiency and the resonance frequency are intrinsically governed by the acoustic resistance and reactance, resulting in near‐perfect absorption. Furthermore, densely matched impedance regions emerge from the seamless integration of diverse component impedances, whereas discrete impedance matching bands are realized by constructing near‐hard boundaries—originating from the coexistence of densely and sparsely distributed resonant modes. In contrast, under backward incidence, the impedance spectra exhibit strong mismatches across the entire frequency range, leading to suppressed mode excitation and yielding near‐total reflection. This unidirectional behavior stems from the asymmetric excitation of non‐Hermitian eigenmodes, where eigenvalue degeneracy occurs only under forward incidence—effectively operating near a unidirectional EP. The corresponding sound pressure distributions at resonant frequencies for both incidence directions are provided in Section S7 (Supporting Information).

In Concept 2, as shown in Figure 5c,d, the BS and SS configurations leverage loss factors switching to achieve bidirectional sound absorption. These systems are designed to decouple the direction‐dependent absorption responses, allowing both incidence directions to independently fulfill the critical coupling condition. The broadband and frequency‐selective responses observed in BS and SS configurations are both supported by impedance matching conditions under forward and backward incidence. Further details are provided in Section S8 (Supporting Information). To elucidate the underlying physical mechanisms, Figure 5e presents the simulated sound pressure distributions at representative frequencies, offering intuitive insight into the active resonant components at each operation state. High‐efficiency sound absorption is achieved through sound energy trapping, with specific resonators contributing to the resonance effect. For instance, under forward incidence, significant sound pressure differences are observed between the two components of the BS configuration within the frequencies of *f*  =  [1065,  1225] Hz, indicating strong localized energy dissipation in the corresponding resonators. In contrast, the remaining two components exhibit negligible pressure variation, suggesting minimal energy conversion. Under backward incidence, three distinct resonance peaks emerge at 395, 765, and 1005 Hz, each dominated by different resonant components. These results demonstrate that sound energy could be selectively trapped and dissipated within specific modular components, governed by the direction of incidence and the resonance characteristics of the structure. This energy trapping behavior arises from the independent tuning of loss factors in specific modular components to access EPs, which generate high sound pressure gradients at their locations. These pressure distributions exhibit mode decoupling, thereby enabling a transition from unidirectional to bidirectional acoustic absorption.

To quantitatively assess the four configurations, Figure 5f summarizes three key performance indicators derived from the absorption spectra in Figure 5a–d. Figure 5f‐i presents the effective bandwidths (defined as the frequency range where the absorption coefficient is greater than or equal to 0.5) for both forward and backward incidence. The maximum effective bandwidth reaches 436 Hz in the B0 configuration under forward incidence, representing a 354% increase relative to that of the SS configuration in the same direction. B0 and S0 configurations exhibit superior forward absorption relative to BS and SS, while their backward absorption remains negligible. In contrast, BS and SS configurations achieve bidirectional absorption. The relatively narrow bidirectional absorption bandwidths observed in BS and SS configurations are primarily due to the division of resonant modes across two incidence directions. However, evaluating acoustic performance based solely on bandwidth is insufficient. Subwavelength low‐frequency absorption is also essential for evaluating the practical viability of acoustic metastructures. Figure 5f‐ii illustrates the wavelength‐to‐structural thickness ratios, highlighting both the structural compactness and low‐frequency efficiency. All configurations operate in the subwavelength regime, with S0 and SS achieving deep‐subwavelength absorption, where the operating wavelengths are ≈36 and 34 times the structural thickness, respectively. Figure 5f‐iii shows the average absorption across the target frequency range of 200–1400 Hz. The B0 and S0 configurations exhibit extreme asymmetry between forward and backward incidences, whereas the BS and SS configurations demonstrate high‐efficiency and well‐balanced absorption in both directions.

These results establish a physical framework that integrates structural resonance hybridization, decoupled asymmetric energy dissipation, and impedance matching modulation. By precisely tailoring the loss factors to access EP and modulating mode coupling characteristics, the system enables a switchable transition between unidirectional and bidirectional absorption states. This approach provides a robust and adaptable strategy for achieving high‐efficiency acoustic wave control in advanced metastructures.

### 3.4 Experimental Implementation

**Figure 6:**
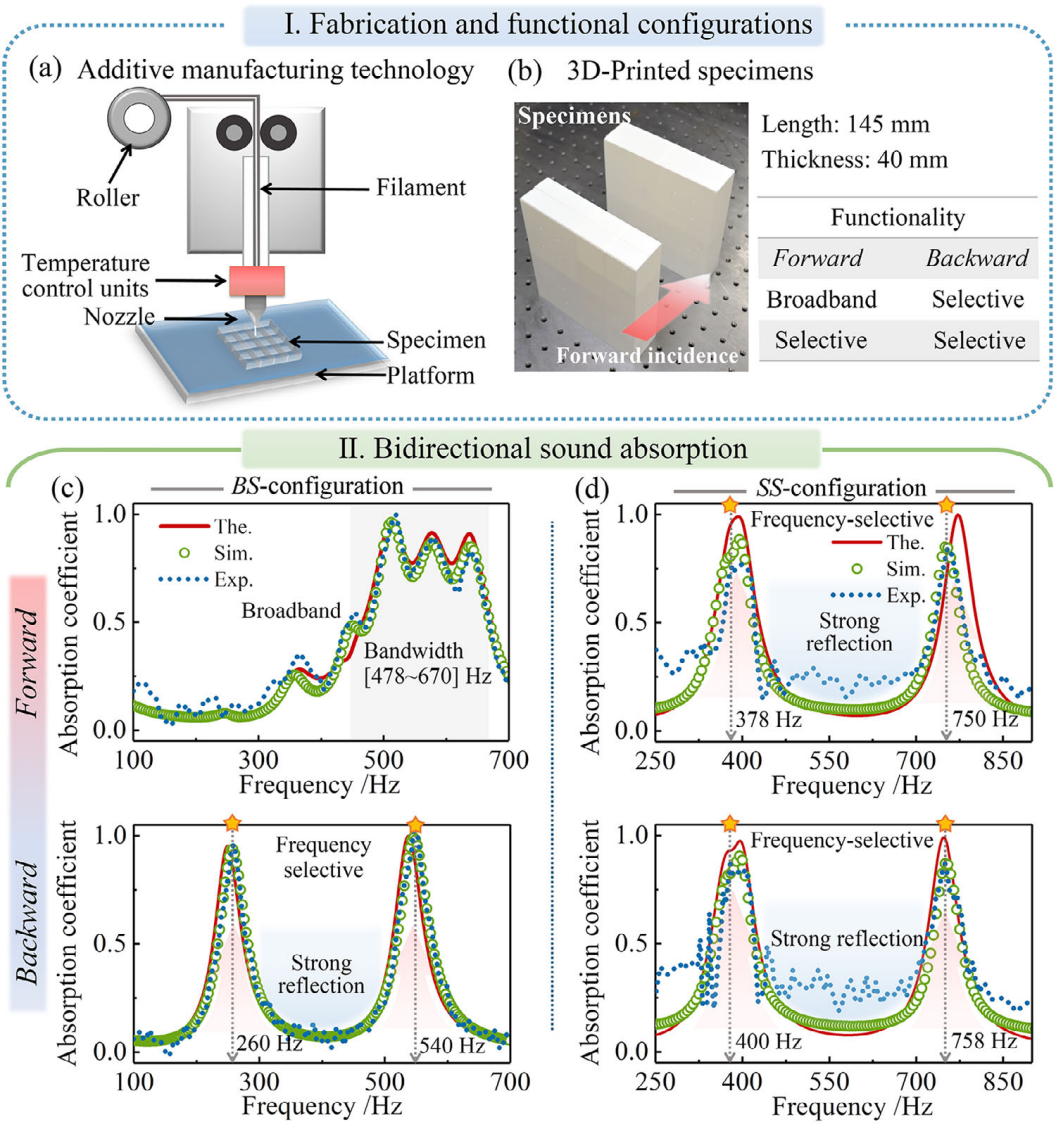
Experimental validation of the proposed metamaterials demonstrating bidirectional broadband and frequency‐selective sound absorption. a) Schematic of the additive manufacturing process based on fused deposition modeling. b) Photographs of the fabricated specimens with a square cross‐section (145 mm × 145 mm) and a total thickness of 40 mm, designed to meet impedance tube testing requirements. c,d) Comparison of sound absorption spectra obtained by theoretical prediction (The.), numerical simulation (Sim.), and experimental measurement (Exp.) for the bidirectional absorption performance.

A switchable bidirectional sound absorption strategy was developed by precisely tuning EPs and integrating interleaved resonator coupling, with its experimental validation shown in **Figure** **6**. Additive manufacturing (AM), commonly known as 3D printing, offers an efficient and precise method for fabricating complex, multiscale metamaterial structures.^[^ ^50^, ^51^, ^52^ ^]^ Recent advances in AM have further enabled the realization of sophisticated acoustic metastructures. Fused deposition modeling (FDM) was selected among various AM techniques for its cost‐effectiveness, material versatility, and widespread accessibility, features that make it well suited for both prototyping and scalable fabrication of acoustic devices.^[^ ^52^, ^53^, ^54^ ^]^ The fabricated structure adopts a Helmholtz‐resonator‐inspired design, consisting of perforated plates and rectangular cavities, and is characterized by relatively simple geometry. Despite the inherent limitations in printing resolution, FDM provides sufficient dimensional accuracy to capture the relevant acoustic behavior and is adequate for experimental validation purposes. The metastructure samples, shown in Figure 6b, were fabricated using FDM 3D printing (Bambu Lab A1 Series) with sufficient dimensional accuracy for acoustic validation. Each sample was designed with a square cross‐section of 145 mm and a thickness of 40 mm to match the impedance waveguide dimensions. The functional performance mapping reveals that the BS configuration exhibits broadband absorption under forward incidence, while it transitions to frequency‐selective absorption under backward incidence. In contrast, the SS configuration maintains frequency‐selective absorption in both directions. The corresponding geometric parameters and experimental setup are detailed in Section S9 (Supporting Information).

Figure 6c,d compares the sound absorption spectra of the proposed metastructures, as obtained from theoretical predictions, numerical simulations, and experimental measurements. The results confirm broadband and frequency‐selective bidirectional absorption performance. The BS configuration exhibits broadband absorption under forward incidence and frequency‐selective absorption under backward incidence, demonstrating clear bidirectional behavior. Under forward incidence, a continuous broadband absorption band spanning 478–670 Hz is achieved, covering a wide low‐frequency range and ensuring efficient energy attenuation. In contrast, backward incidence yields two discrete absorption peaks at 260 and 542 Hz, corresponding to effective absorption in the low and mid‐frequency ranges. These responses exhibit deep‐subwavelength characteristics, with the structural thickness ranging from ≈1/33 to 1/15.8 of the corresponding wavelengths (λ  = *c*_0_/*f* , where λ represents the wavelength).

For the second design targeting bidirectional frequency‐selective absorption, nearly identical absorption peaks are observed from both sides, with resonance frequencies appearing at 378 and 750 Hz under forward incidence, and at 400 and 758 Hz under backward incidence. Since the proposed design is based on resonance‐driven sound absorption, accurate prediction of peak frequencies, amplitudes, and effective bandwidths is crucial for evaluating model reliability. The close agreement among theoretical, numerical, and experimental results confirms both the validity of the simulation framework and the robustness of the proposed switchable bidirectional absorption strategy. A detailed quantitative comparison illustrating these consistencies is presented in Section S10 (Supporting Information). To further interpret minor discrepancies in peak values and bandwidths, Section S11 (Supporting Information) investigates the role of microgeometric imperfections introduced during FDM fabrication, such as pore edge deformation and surface roughness, which affect viscous dissipation and resonance behavior.

Building on this validated framework, the proposed design incorporates multi‐scale coupling to modulate direction‐dependent acoustic responses. Each hierarchical component contributes distinct resonances, and their strategic arrangement enhances both bandwidth and coupling strength. This design strategy enables efficient bidirectional sound absorption and offers a practical solution for compact, broadband, and low‐frequency noise control.

## 4 Scientific Advance, Application, and Generalized Framework

The proposed interleaved coupling and EP modulation strategy establishes a novel design paradigm for acoustic metastructures, particularly in achieving tunable bidirectional absorption. It effectively overcomes the long‐standing limitations of conventional resonator‐based systems, which are typically constrained to unidirectional absorption with limited tunability. This approach provides a comprehensive theoretical and physical framework for realizing switchable, bidirectional sound absorption in integrated acoustic systems.

**Figure 7:**
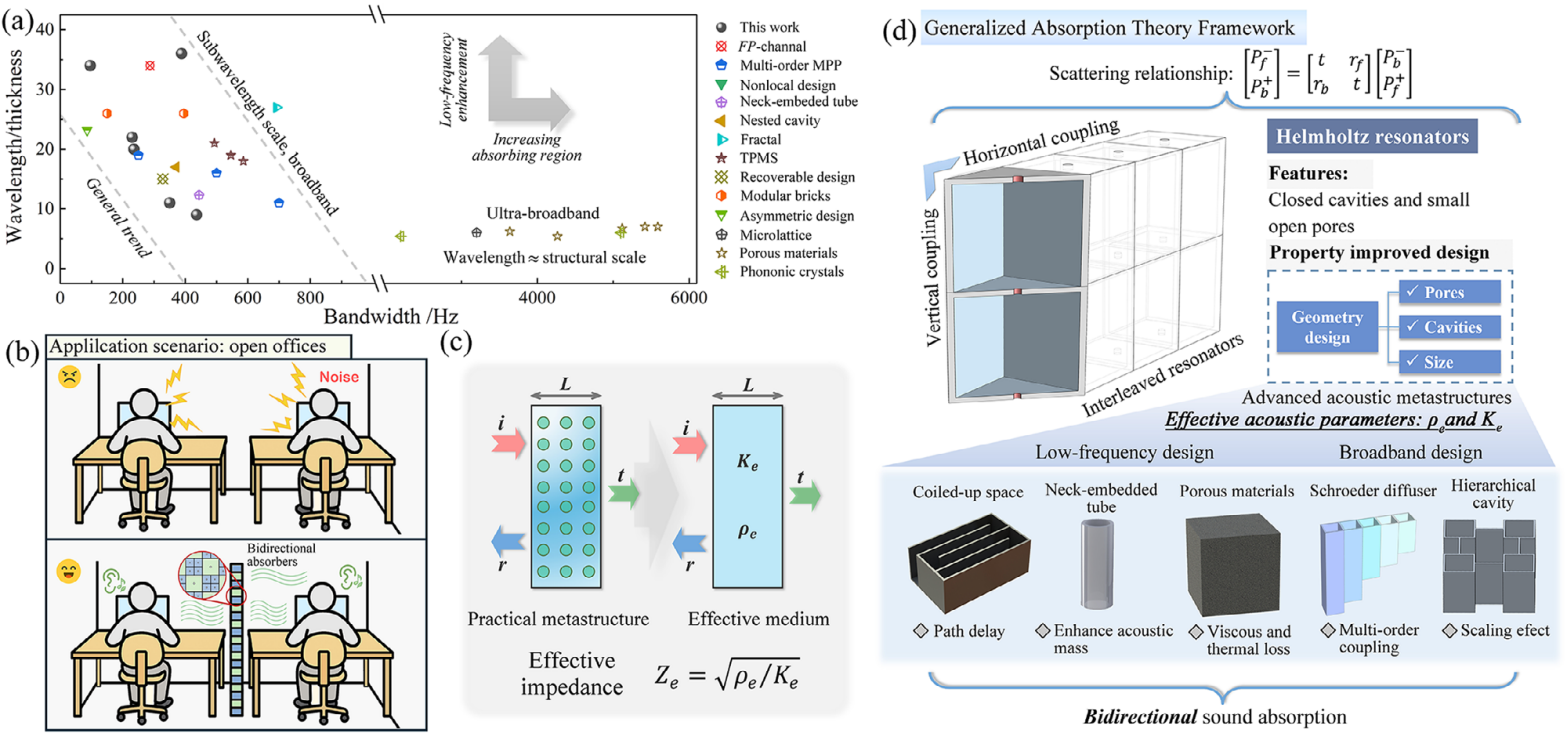
Comparative performance, application prospects, and generalized theoretical framework. a) Benchmark map illustrating the tradeoff between effective sound absorption bandwidth and low‐frequency performance, compared with various existing advanced designs including FP‐channel,^[^ ^21^ ^]^ multi‐order MPP,^[^ ^57^ ^]^ nonlocal design,^[^ ^16^ ^]^ neck‐embedded tube,^[^ ^58^ ^]^ nested cavity,^[^ ^22^ ^]^ fractal,^[^ ^59^ ^]^ TPMS,^[^ ^60^ ^]^ recoverable design,^[^ ^14^ ^]^ modular bricks,^[^ ^19^ ^]^ asymmetric design,^[^ ^34^ ^]^ micro lattice,^[^ ^15^ ^]^ porous materials^[^ ^11^ ^]^ and phononic crystals.^[^ ^61^ ^]^ b) Application scenario in open‐plan offices where bidirectional absorbers effectively mitigate mutual noise disturbance between users. c) Schematic illustration of the effective medium theory for resonant acoustic metastructures. d) Generalized absorption theory framework for resonance‐based metastructures. This modal highlights the design of advanced acoustic metastructures capable of achieving bidirectional low‐frequency or broadband absorption by modulating the effective density ρ_*e*_ and bulk module*K_e_* within the theoretical framework developed in this work.

**Figure** **7**a presents a bandwidth versus wavelength‐to‐thickness ratio map, highlighting the dual advantages of the proposed design: broadband absorption and deep‐subwavelength structural compactness, both essential for effective low‐frequency sound control.^[^ ^55^ ^]^ In this context, the absorption bandwidth represents the system's overall energy dissipation capacity, whereas the wavelength‐to‐thickness ratio indicates structural compactness and effective performance in the low‐frequency regime. The proposed metastructure, exhibiting robust bidirectional absorption, achieves comparable or even superior performance to classical and advanced acoustic absorbers, including Fabry–Pérot channels, porous media, nested cavities, and nonlocal or topological configurations, most of which remain constrained to unidirectional operation. Beyond laboratory demonstrations, Figure 7b illustrates a real‐world application scenario in an open‐plan office environment. Unlike traditional absorbers that require directional alignment or full‐surface coverage, the proposed design enables bidirectional, high‐efficiency sound energy dissipation, preserving architectural openness while effectively mitigating mutual acoustic disturbances between users.

Notably, for resonant acoustic metastructures, the effective medium theory provides a powerful theoretical framework to characterize their macroscopic acoustic behavior. Its core principle lies in approximating locally inhomogeneous structures as acoustically homogeneous media with equivalent macroscopic properties, particularly in the long‐wavelength regime.^[^ ^56^ ^]^ In such resonant systems, the resonance wavelengths are much larger than the physical dimensions of the unit cells, making the homogenization approach especially appropriate and insightful. The effective parameters can be extracted using the inverse parameter retrieval method, by matching the transmission and reflection coefficients of the metastructure to those of an equivalent homogenized medium. As shown in Figure 7c, the real metastructure configuration can be replaced by an equivalent uniform layer that yields the same transmission and reflection coefficients. The effective specific acoustic impedance is then determined as $Z_{e}=\sqrt{\rho_{e}/K_{e}}$, where ρ_*e*_ and *K_e_* are the effective density and bulk modulus, respectively. This impedance can then be used to quantitatively evaluate the sound absorption performance of the system.

Building on this foundation, we extend the impedance‐based formulation to a generalized framework for bidirectional absorption design. As illustrated in Figure 7d, this strategy enables application across a broader class of resonator‐based acoustic systems. A typical HR consists of a closed cavity and an open pore. When incident sound waves enter through the pore, resonance occurs, enabling impedance matching with the surrounding medium. This principle is also applicable to more complex geometries. To achieve superior acoustic absorption, numerous advanced metastructures have been developed by optimizing and modifying geometrical configurations, such as coiled‐up channels, neck‐embedded tubes, porous materials, Schroeder diffusers, and hierarchical cavities. While structurally diverse, these designs share a unified underlying mechanism, the modulation of effective acoustic parameters, particularly effective density and bulk modulus, which define the specific impedance and govern resonance behavior. This modulation enables enhanced absorption in both lower‐frequency and broadband regimes.

The present design is established within an impedance modulation framework, which is further enriched through the integration of non‐Hermitian EP physics and interleaved resonator coupling. EPs introduce eigenvalue/ eigenmode coalescence and enable asymmetric absorption responses under asymmetric loss, thus supporting controllable transitions between unidirectional and bidirectional absorption states. This physical insight not only enhances absorption tunability in the current design but also serves as the foundation for a broadly applicable bidirectional absorption strategy. Although the proposed metastructure demonstrates efficient absorption within the subwavelength range above 200 Hz, the underlying framework is inherently scalable and not limited to the current Helmholtz‐resonator‐based design. The bidirectional absorption strategy is broadly applicable to a wide range of resonant acoustic metastructures and holds strong potential for achieving deep‐subwavelength, low‐frequency performance. More importantly, the EP‐guided impedance‐based strategy proposed here is not confined to the specific structure demonstrated. When other resonance‐based or porous absorbers are reconfigured as two‐port acoustic systems, their behavior can be governed by similar impedance conditions and EP modulation. This establishes a unified theoretical and design approach for achieving robust, broadband, and bidirectional sound absorption across a wide class of acoustic metastructures.

## 5 Conclusion

In summary, this work presents a physics‐driven strategy for achieving robust and switchable bidirectional sound absorption by integrating interleaved resonator coupling with exceptional point modulation. Through coordinated control of vertical and horizontal resonance interactions, the proposed metastructures enable both broadband and frequency‐selective absorption under forward and backward incidence, all within a compact geometry. Theoretical analysis, numerical simulation, and experimental validation collectively confirm their high‐efficiency bidirectional performance, effectively overcoming the limitations of conventional unidirectional absorbers. Moreover, by extending the EP‐based impedance modulation framework to other resonator‐based metamaterials, a generalized design paradigm is proposed to facilitate the transformation from unidirectional to bidirectional absorption.

While the current design demonstrates promising acoustic performance, it also presents several practical trade‐offs, including increased structural complexity and challenges in large‐scale fabrication due to the multiscale hierarchical geometry. Future work will focus on developing modular, reconfigurable, or tunable resonator systems to simplify fabrication and enhance adaptability across different frequency ranges and application scenarios. Overall, these insights not only expand the design versatility of acoustic metastructures but also lay a foundation for scalable, high‐performance solutions in next‐generation noise‐control technologies.

## 6 Experimental Section

### Numerical Simulations

Numerical simulations were conducted in COMSOL Multiphysics by coupling the pressure acoustics and thermoviscous acoustics modules. As detailed in Section S1 (Supporting Information), sound wave propagation is governed by the pressure acoustics module, with the background medium impedance set to  *Z*_0_ =  1.2 kg/*m* ^3^ × 343 m/s. To accurately capture thermal and viscous losses within sub‐millimeter perforations, the thermoviscous acoustics module is additionally employed. The TMM is employed to calculate the reflection, transmission, and absorption coefficients. Microphones Mic S1 and Mic S2 are positioned to record incident and reflected pressure signals, respectively. The transmission coefficient is obtained by evaluating the ratio of transmitted to incident sound power.

### Fabrication

The acoustic metastructures were fabricated using a commercial FDM 3D printer (Bambu Lab A1 Series) equipped with a brass nozzle of 0.4 mm diameter. Polylactic acid (PLA) filament was selected as the printing material. The printing process was conducted at a nozzle temperature of 215 °C and a build platform temperature of 60 °C. A layer height of 0.1 mm was used to ensure high geometric fidelity and structural stability. The final printed structure maintained the designed dimensions with minimal deviation, enabling accurate experimental validation of its acoustic performance.

### Experiments

Experimental measurements were conducted using the two‐load method in accordance with ASTM E2611‐17. The measurement setup is detailed in Section S9 (Supporting Information). The specimen was mounted inside a waveguide with a square cross‐section. A loudspeaker positioned at one end of the tube generated white noise, which was amplified using a Brüel & Kjær (B&K) Type 2734‐A power amplifier. The opposite end of the tube was terminated with either a rigid backing or an absorbing sponge. Four 1/4‐inch B&K Type 4494‐A microphones were placed at designated positions along the tube to record the acoustic pressure fields. A LAN‐XI Light 4‐channel data acquisition module (Type 3677) was employed to measure the transfer functions between microphones, enabling the calculation of absorption coefficients. To ensure measurement reliability, each sample was tested three times under identical conditions, and the results showed excellent reliability. Minor discrepancies between numerical and experimental results are primarily attributed to geometric imperfections arising from FDM printing, such as pore edge deformation and surface roughness, which are further analyzed in Section S11 (Supporting Information).

## Conflict of Interest

The authors declare no conflict of interest.

## Supporting information



Supporting Information

## Data Availability

The data that support the findings of this study are available from the corresponding author upon reasonable request.
