# Supplementary material for: Switchable Bidirectional Sound Absorption Via Exceptional Point Modulation in Acoustic Metastructures with Interleaved Resonator Coupling
Source: Adv Sci (Weinh). 2025 Aug 11;12(44):e08951. doi: 10.1002/advs.202508951 (PMC12667512; doi:10.1002/advs.202508951)
Supplement: Supplementary file 1 — Supporting Information [file ADVS-12-e08951-s001.docx]

Supporting Information

**Switchable Bidirectional Sound Absorption via Exceptional Point Modulation in Acoustic Metastructures with Interleaved Resonator Coupling**

*Zichao Guo, Liangfen Du, Zirui Yang, Kexin Zeng, Ziping Lei, Zhonggang Wang^*^, Zhendong Li ^*^, Zheng Fan^*^*

## Section S1. Numerical simulation method


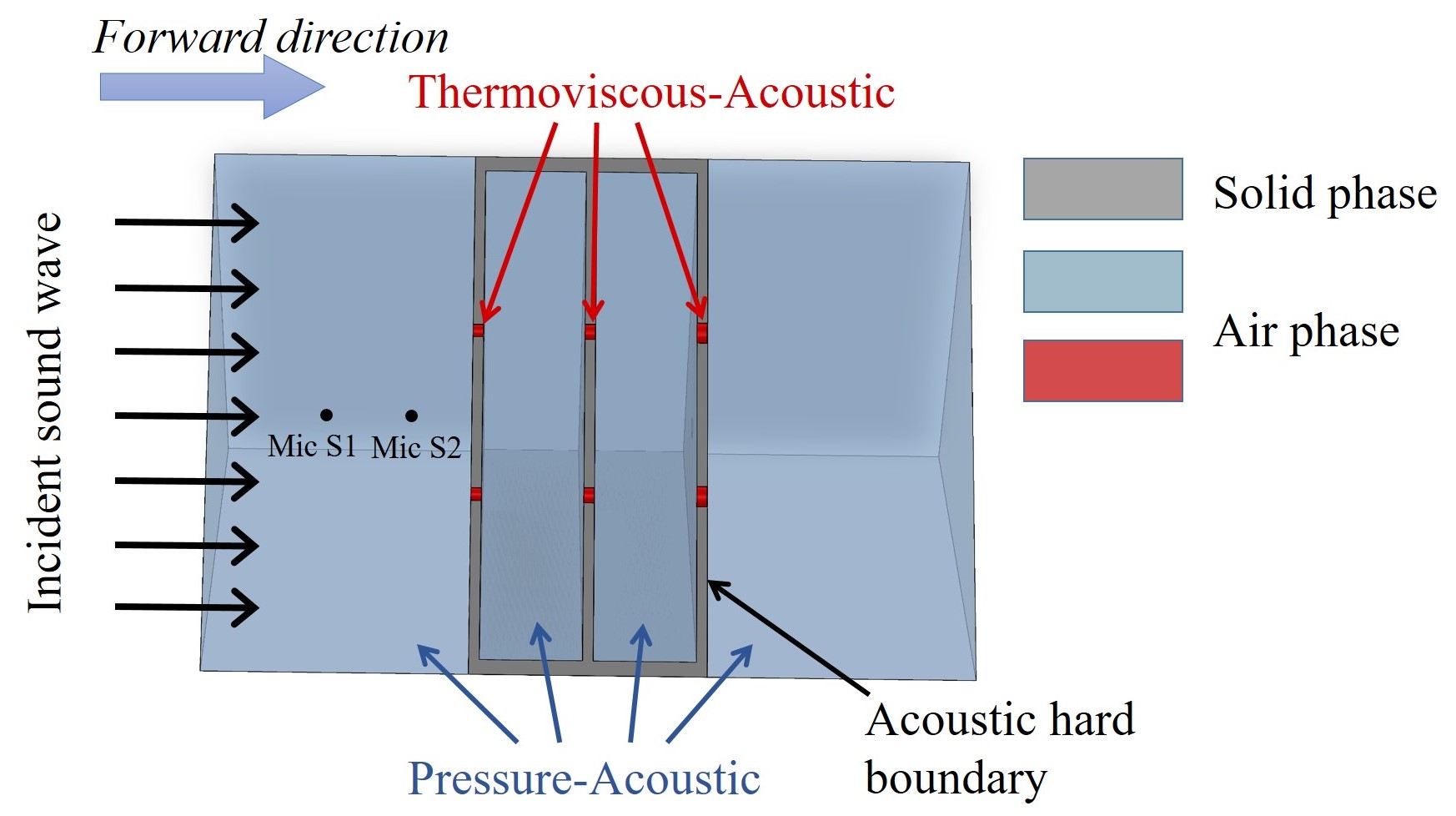


**Figure S1.** Schematic of the finite element model implemented in COMSOL Multiphysics. Red and blue regions denote the air domains within the pores and cavities, respectively, while the solid frame is shown in gray. The propagation of sound waves is governed by the pressure-acoustics module. Due to the impedance mismatch between air and solid regions, the air–solid interfaces (gray areas) are modeled as acoustically hard boundaries. Furthermore, due to the sub-millimeter pore dimensions and associated thermal and viscous losses, the thermoviscous acoustics module is incorporated for enhanced accuracy ^[S1]^.

## Section S2. Sound pressure field distributions

To illustrate the mode behavior underlying the observed dual-peak absorption, **Figure S2** presents the simulated sound pressure field distributions at the two resonant frequencies. At $f_{Peak1}=255 \mathrm{Hz}$, the sound pressure is predominantly concentrated within the lower cavity, indicating strong energy localization. In contrast, at $f_{Peak2}=637 \mathrm{Hz}$, the pressure distribution shifts toward the upper cavity, which exhibits greater amplitude variation. In both cases, a sharp pressure gradient emerges across the internal connecting hole, revealing a region of enhanced local energy dissipation. These results further confirm that the internal hole serves not only to connect the two cavities in series, but also introduces a phase delay and partial reflection. This mechanism enables frequency-dependent energy dissipation, thereby supporting the observed two-resonance absorption behavior.


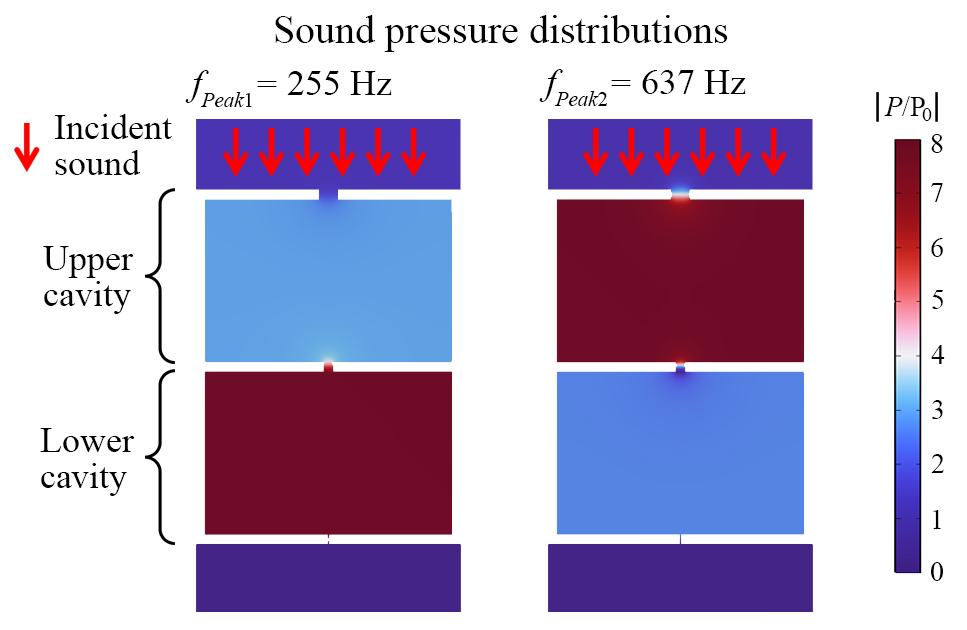


**Figure S2.** Sound pressure distributions when sound waves radiate from the forward side.

## Section S3. Effect of Internal Hole Size on Absorption Performance

Adjusting the size of the internal hole allows effective tuning of energy dissipation and acoustic coupling between the two cavities. Such tuning affects the critical coupling condition, thereby enabling bidirectional or asymmetric absorption behavior. A parametric analysis is conducted by varying the internal hole diameter $d_{2,1}$, while all other geometric parameters are held constant. As illustrated in **Figure S3**, the absorption coefficient spectra shift significantly with different values of $d_{2,1}$​. Notably, an optimum coupling state is observed at $d_{2,1}=1.135 mm$ (red curve), where quasi-perfect absorption occurs at the target frequency. For over-coupled or under-coupled conditions, the absorption performance deteriorates due to an imbalance between external radiation loss and internal dissipation. These results highlight the critical role of inter-pore coupling strength in tailoring the absorption response of the system.


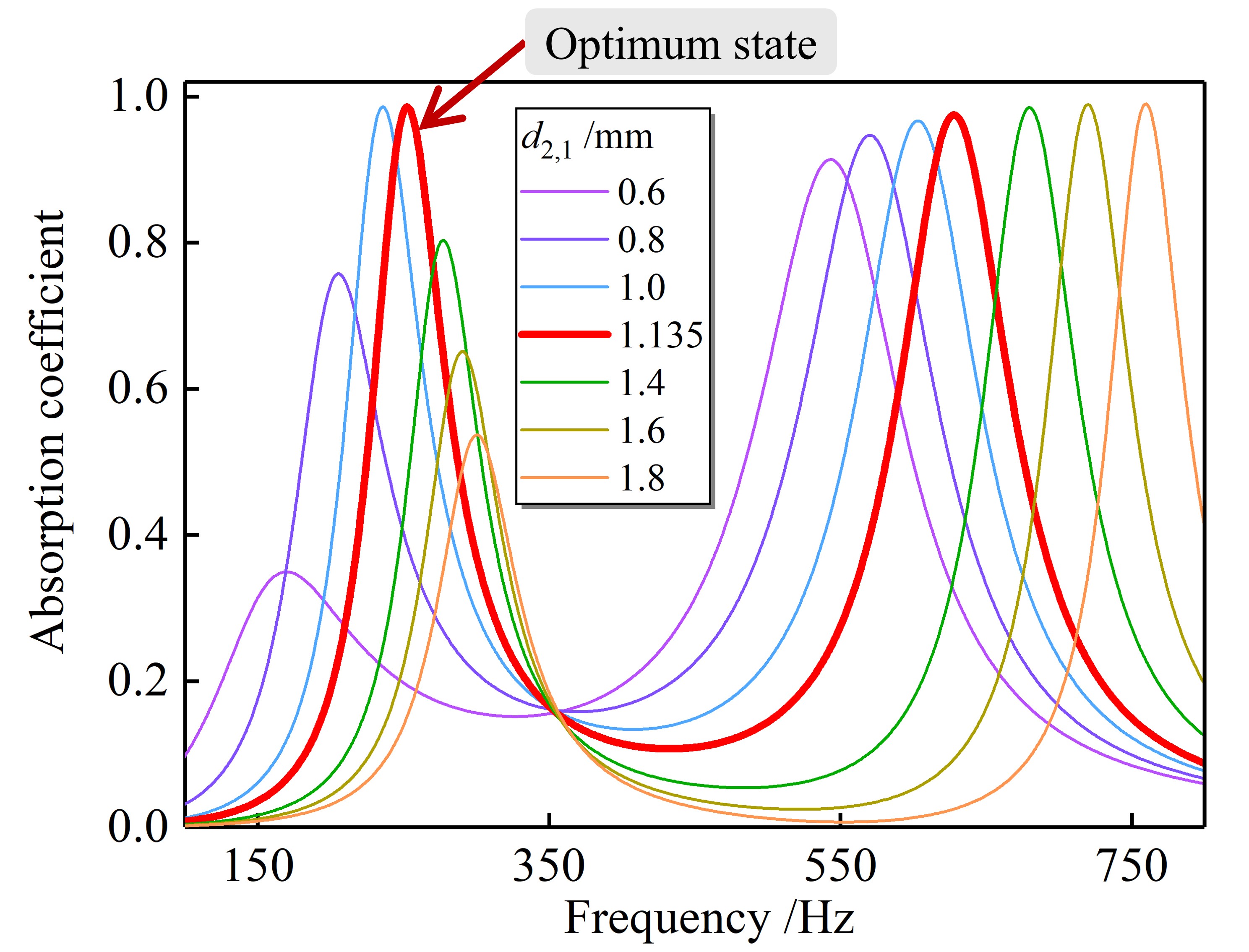


**Figure S3.** Sound-absorption performance of different loss factors $d_{2,1}$ represented by the colored lines.

## Section S4. Damping-state analysis via complex frequency plane

The absorbing behavior can be expressed in the distributions of ${\log_{10} \left| r \right|}^{2}$ in the complex frequency plane, where the angular frequency is represented as $\omega=\omega_{r}+j\omega_{i}$^[S2, S3]^. This representation reveals the position and movement of system zeros (blue dots) and poles (red triangles) under different damping conditions, offering insights into impedance matching and energy dissipation behavior, as shown in **Figure S4**. In the lossless case, the system exhibits a pair of complex-conjugate poles and zeros symmetrically located across the real axis.

As loss is introduced, the zero moves toward the real axis. When the zero remains in the upper half-plane, the intrinsic loss is smaller than the leakage loss, resulting in insufficient energy dissipation and an under-damped state. Upon increasing the loss such that the zero lands precisely on the real axis, the system reaches a critical coupling condition, where the intrinsic loss exactly balances the leakage. This yields perfect absorption and optimal impedance matching. Further increasing the loss shifts the zero into the lower half-plane, corresponding to an over-damped regime. In this case, the excessive internal loss breaks the impedance matching with the background medium (e.g., air), leading to enhanced reflection and reduced absorption. Therefore, analyzing the evolution of zeros and poles in the complex frequency domain provides a clear physical interpretation of how damping states govern the absorption performance of resonant acoustic systems.


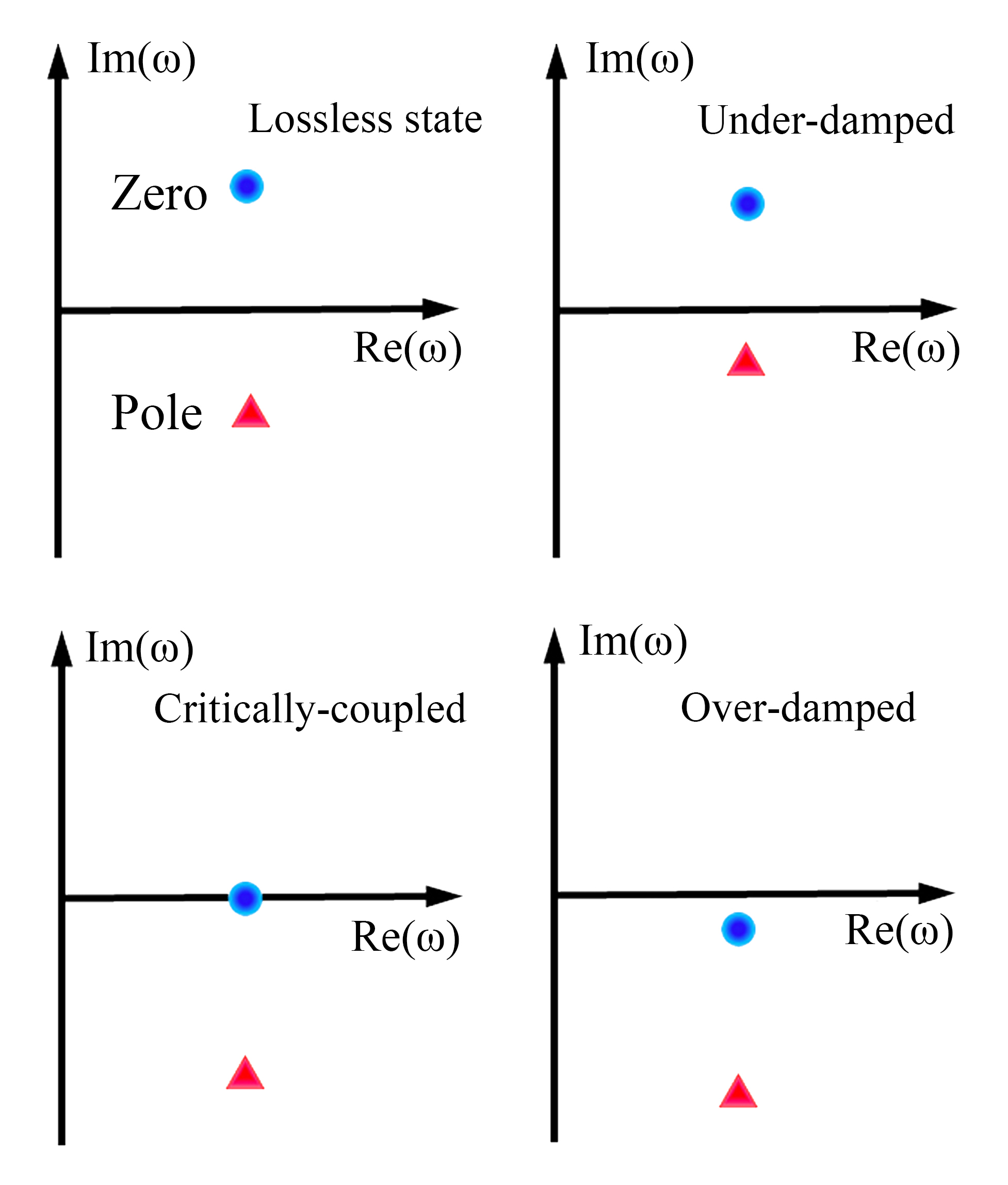


**Figure S4.** Complex frequency plane representation of the reflection coefficients.

## Section S5. Influence of sound absorption performance via tuning length, and thickness as the loss factors


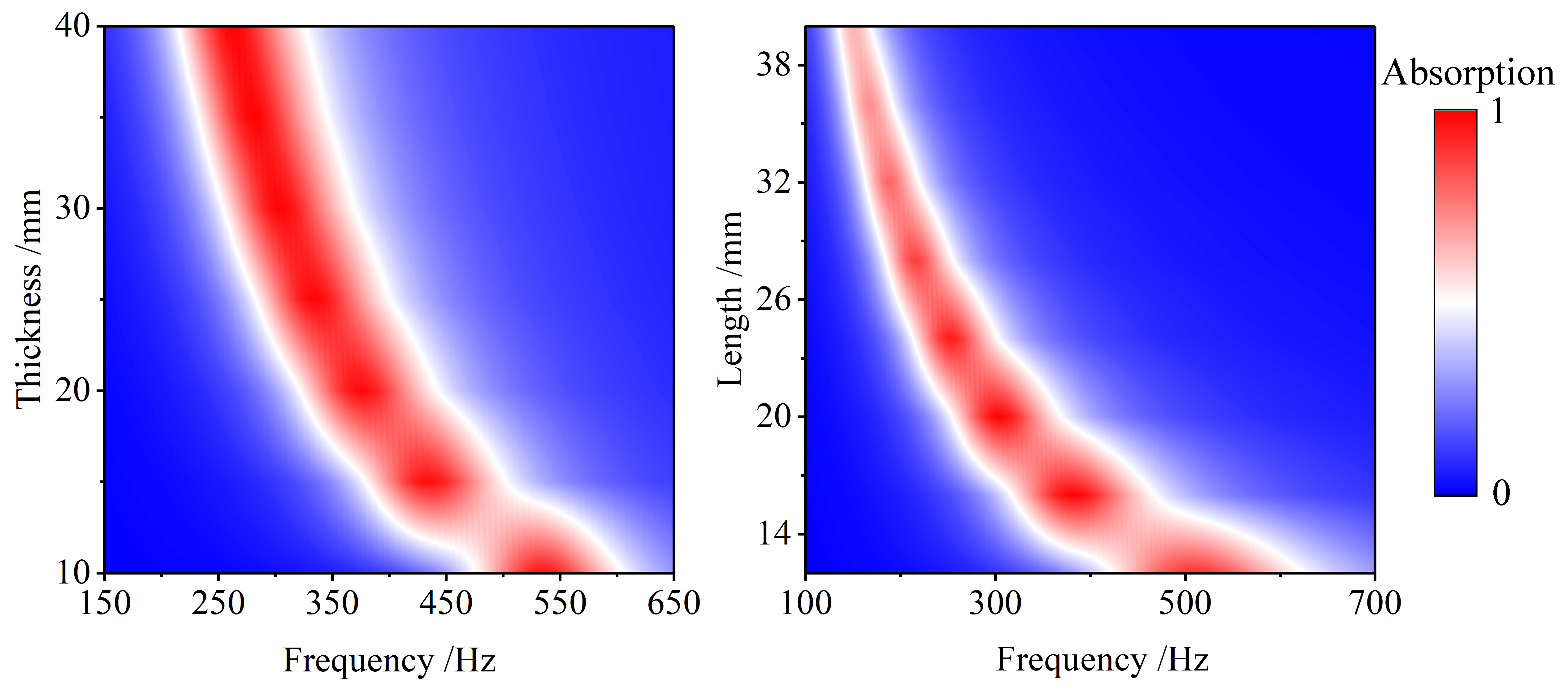


**Figure S5.** Acoustic performance of the structure by varying its geometric parameters. An increase in the structural thickness results in a shift of the absorption resonance toward lower frequencies, indicating an inverse relationship between thickness and resonance frequency. The right panel demonstrates that increasing the planar dimension of the unit also leads to lower resonance frequencies. In both cases, the red regions correspond to near-unity absorption (Absorption $\approx$ 1), confirming that tailored geometry enables precise control over the working frequency range. These results demonstrate the feasibility of achieving application-specific acoustic performance through basic geometric modifications.

## Section S6. Geometric configurations and parameters of unidirectional and bidirectional performance

**Table S1** Geometric parameters of validation model. (Unit: mm)

| $l_{i}$ | $h_{1}$ | $h_{2}$ | $t_{1}$ | $t_{2}$ | $t_{3}$ | $d_{1,1}$ | $d_{2,1}$ | $d_{3,1}$ |
| --- | --- | --- | --- | --- | --- | --- | --- | --- |
| 28 | 18.5 | 18.5 | 1.0 | 1.0 | 1.0 | 2.0 | 1.25 | 0.2 |
| 13.5 | 18.5 | 18.5 | 1.0 | 1.0 | 1.0 | 1.5 | 0.8 | 0.35 |

**Table S2** Geometric parameters of broadband and zero absorption performance. (Unit: mm)

| $l_{i}$ | $h_{1}$ | $h_{2}$ | $t_{1}$ | $t_{2}$ | $t_{3}$ | $d_{1,1}$ | $d_{2,1}$ | $d_{3,1}$ |
| --- | --- | --- | --- | --- | --- | --- | --- | --- |
| 13.5 | 18.5 | 18.5 | 1.0 | 1.0 | 1.0 | 1.5 | 0.7 | 0.4 |
| 6.25 | 18.5 | 18.5 | 1.0 | 1.0 | 1.0 | 0.8 | 0.3 | 0.25 |

**Table S3** Geometric parameters of frequency-selective and zero absorption performance. (Unit: mm)

| $l_{i}$ | $h_{1}$ | $h_{2}$ | $t_{1}$ | $t_{2}$ | $t_{3}$ | $d_{1,1}$ | $d_{2,1}$ | $d_{3,1}$ |
| --- | --- | --- | --- | --- | --- | --- | --- | --- |
| 28 | 18.5 | 18.5 | 1.0 | 1.0 | 1.0 | 2.2 | 1.0 | 0.25 |
| 13.5 | 18.5 | 18.5 | 1.0 | 1.0 | 1.0 | 1.5 | 1.0 | 0.4 |

**Table S4** Geometric parameters of frequency-selective and frequency-selective absorption performance. (Unit: mm)

| $l_{i}$ | $h_{1}$ | $h_{2}$ | $t_{1}$ | $t_{2}$ | $t_{3}$ | $d_{1,1}$ | $d_{2,1}$ | $d_{3,1}$ |
| --- | --- | --- | --- | --- | --- | --- | --- | --- |
| 28 | 18.5 | 18.5 | 1.0 | 1.0 | 1.0 | 2.0 | 1.25 | 0.2 |
| 13.5 | 18.5 | 18.5 | 1.0 | 1.0 | 1.0 | 0.35 | 0.8 | 1.5 |

**Table S5** Geometric parameters of broadband and frequency-selective absorption performance. (Unit: mm)

| $l_{i}$ | $h_{1}$ | $h_{2}$ | $t_{1}$ | $t_{2}$ | $t_{3}$ | $d_{1,1}$ | $d_{2,1}$ | $d_{3,1}$ |
| --- | --- | --- | --- | --- | --- | --- | --- | --- |
| 13.5 | 18.5 | 18.5 | 1.0 | 1.0 | 1.0 | 1.7 | 0.9 | 1.2 |
| 13.5 | 18.5 | 18.5 | 1.0 | 1.0 | 1.0 | 0.2 | 1.0 | 1.2 |
| 6.25 | 18.5 | 18.5 | 1.0 | 1.0 | 1.0 | 1.0 | 0.2 | 0.2 |

## Section S7. Sound pressure distribution of unidirectional sound absorption


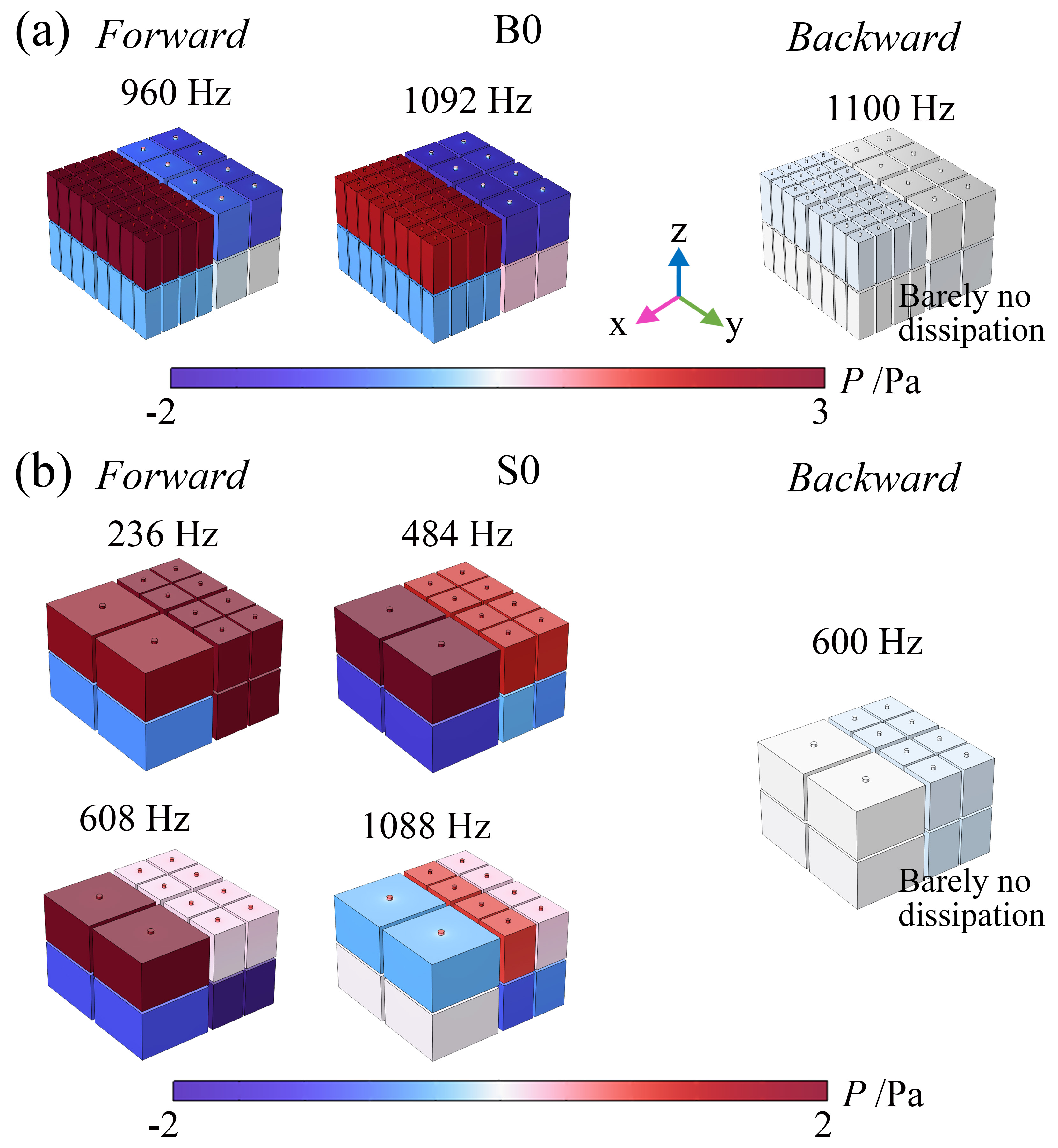


**Figure S6.** Sound pressure distributions at the resonance frequencies for the B0 and S0 configurations. (a) Significant sound pressure differences are observed at 960 Hz and 1092 Hz, respectively, indicating highly efficient energy dissipation. In contrast, backward incidence shows negligible pressure variation, demonstrating the unidirectional sound absorption behavior. (b) Four discrete resonance frequencies exhibit discrete absorption peaks with strong dissipation under forward incidence. Conversely, backward incidence results in minimal dissipation, confirming the near-zero sound absorption performance.

## Section S8. Acoustic impedance analysis of bidirectional sound absorption


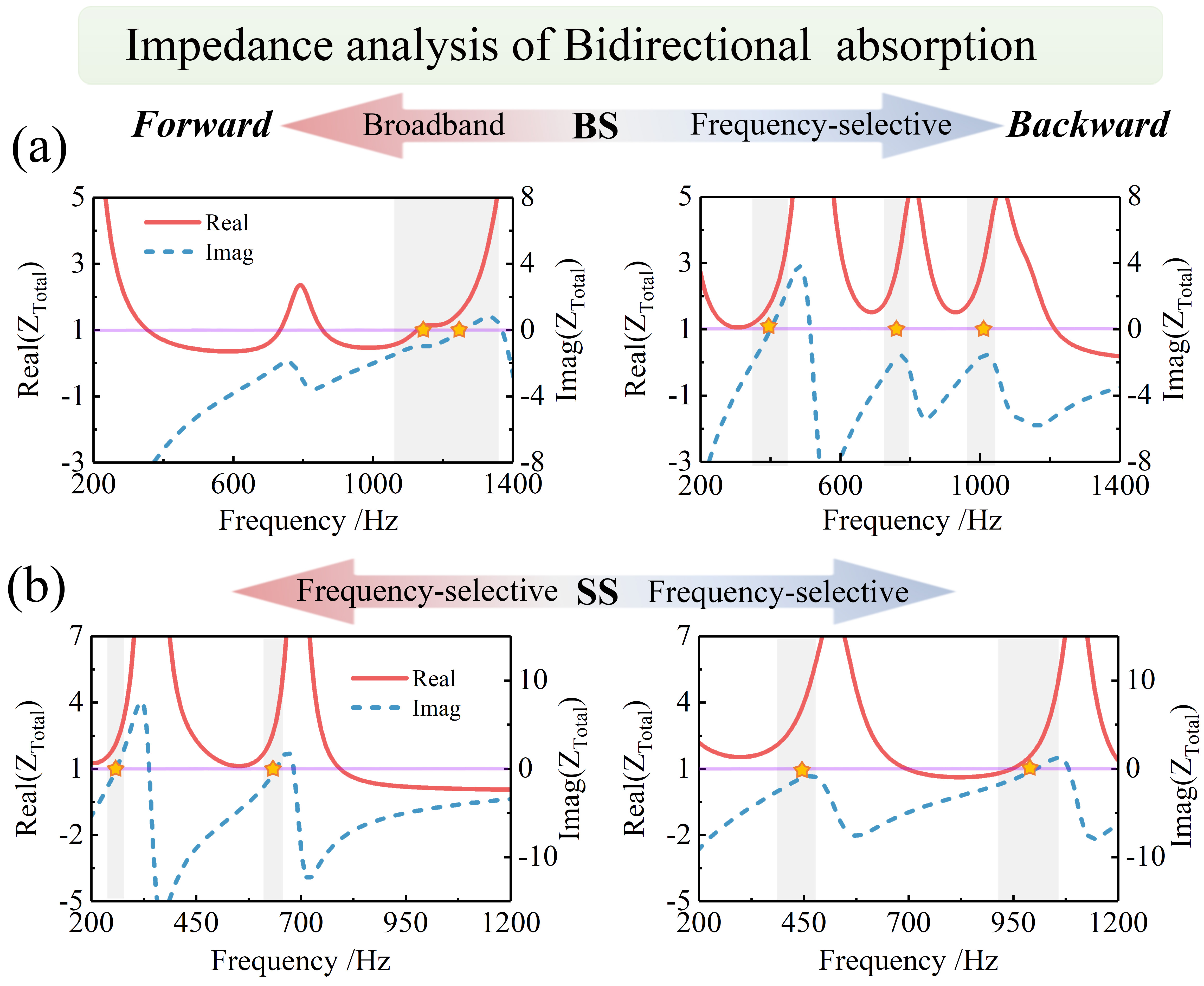


**Figure S7.** Acoustic impedance analysis of bidirectional sound absorption. For the BS case (a), the forward incidence shows a broad impedance-matched region, where the real part of the total impedance approaches unity and the imaginary part crosses zero at multiple points, indicating a broadband absorption regime. Under backward incidence, discrete resonance peaks correspond to localized impedance matching, supporting frequency-selective absorption. For the SS configuration (b), both forward and backward directions exhibit symmetric impedance profiles with well-aligned real and imaginary parts at the resonance frequencies. These results validate the bidirectional absorption behavior of the BS and SS structures, governed by direction-dependent mode coupling and impedance tuning.

## Section S9. Sound absorption measurements

The experiment is carried out in a rectangular waveguide made of acrylic plate. The cross-section of the waveguide is $145 \mathrm{mm} \times145 mm$. A loudspeaker positioned at one end of the tube generated white noise, which was amplified using a Brüel & Kjær (B&K) Type 2734-A power amplifier, generates the incident waves in the waveguide. Four 1/4-inch condenser microphones (B&K Type 4494-A) are used to measure the sound pressure at corresponding positions according to the requirements of transfer function method. The data is collected by A LAN-XI Light 4-channel data acquisition module (Type 3677). The sound absorption coefficients were measured in a rectangular acrylic waveguide using four microphones and a digital frequency analysis system, following the ASTM E2611-17 standard for impedance and absorption testing. The schematic of the impedance tube is shown in **Figure S8**. The impedance tube tests in this study were conducted using a custom-built tub, rather than a commercial system. Therefore, the frequency limits are calculated based on the microphone spacing and tube diameter according to ASTM E2611-17 ^[S4]^

|  | $f_{l}=\frac{1\%c}{s},f_{u}=\frac{50\%c}{d}.$ | (S1) |
| --- | --- | --- |

where $c=343 m/s$ is the speed of sound, $s=50 mm$ is spacing between microphones, and $d=145 mm$ is the side size of tube. This gives a valid measurement frequency range of $68.6 Hz<f<1182.8\mathrm{Hz}$. To characterize the bidirectional absorption performance under identical conditions, the sample was flipped in orientation within the waveguide to allow sound incidence from both the front and back sides. This symmetric testing approach ensures consistent boundary conditions while capturing direction-dependent absorption features. The absorption coefficient of normal incidence can then be obtained ^[S5-S7]^.

Note that the pictures of specimens and setup in this work were taken with a *Nikon Z6 Ⅱ* camera.

**Table S6** Geometric parameters of experimental specimens. (Unit: mm; notation such as “2.2 × 4” denotes four holes each with a diameter of 2.2 mm)

| Specimens | $l_{i}$ | $h_{1}$ | $h_{2}$ | $t_{1}$ | $t_{2}$ | $t_{3}$ | $d_{1,1}$ | $d_{2,1}$ | $d_{3,1}$ |
| --- | --- | --- | --- | --- | --- | --- | --- | --- | --- |
| BS  configuration | 70 | 18.5 | 18.5 | 1.0 | 1.0 | 1.0 | $2.2\times4$ | $2.2\times2$ | 1.2 |
|  | 34 | 18.5 | 18.5 | 1.0 | 1.0 | 1.0 | $2.0\times2$ | 1.2 | 2.0 |
| SS  configuration | 70 | 18.5 | 18.5 | 1.0 | 1.0 | 1.0 | $2.0\times4$ | 1.0 | 0.8 |
|  | 34 | 18.5 | 18.5 | 1.0 | 1.0 | 1.0 | $2.0\times2$ | 2.0 | 1.0 |


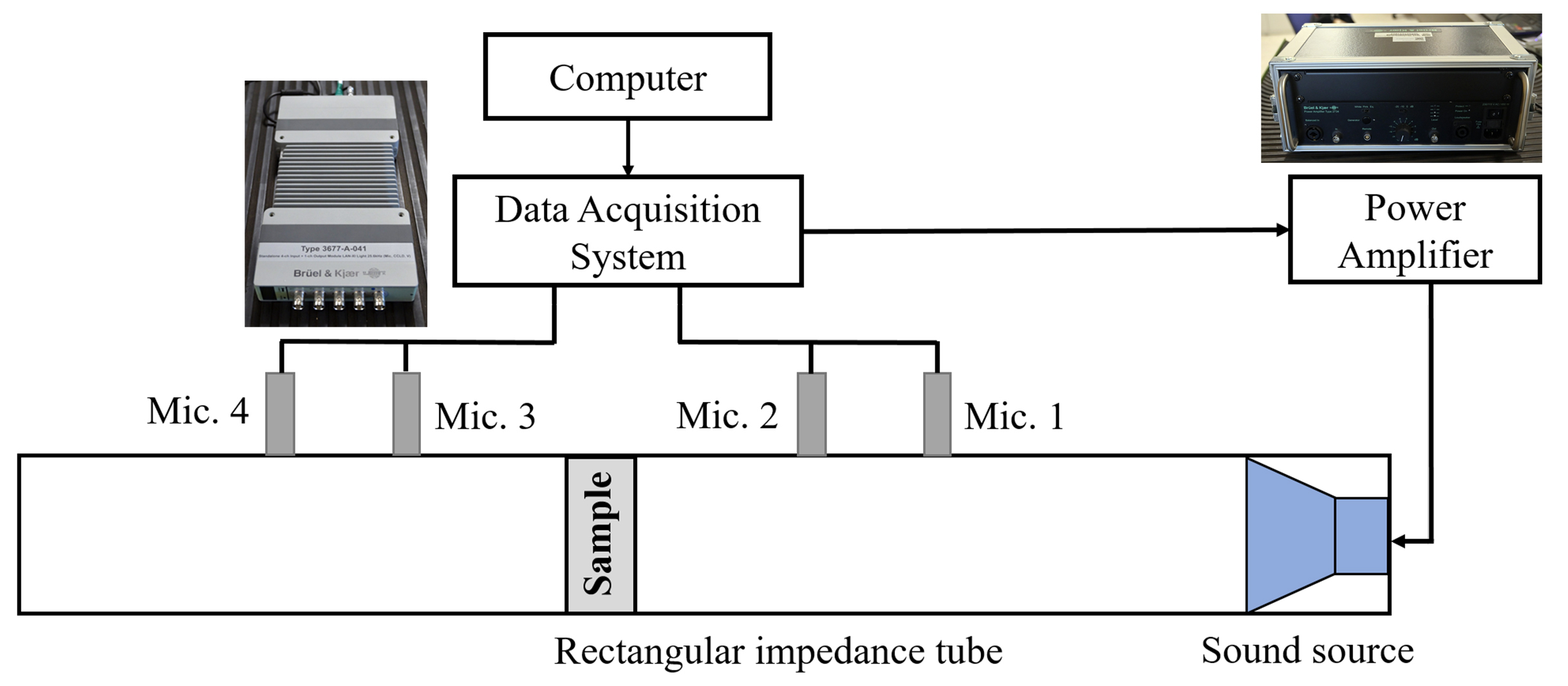


**Figure S8.** Schematics of the impedance waveguide.

## Section S10. Quantitative comparison of absorption performance across theory, simulation, and experiment.

Since the proposed design relies on resonance-driven sound absorption, the accurate prediction of peak frequencies, absorption amplitudes, and effective absorption bandwidths is critical for evaluating the reliability of the theoretical and numerical models. **Figure S9** provides a quantitative comparison of absorption performance among theoretical predictions, numerical simulations, and experimental measurements. Excellent agreement is observed, particularly in the resonance peak locations and magnitudes, highlighting the validity of the simulation framework and the reproducibility of the experimental procedure. The observed deviations are primarily attributed to geometric imperfections introduced during the FDM fabrication process, which slightly affect the effective pore size and structural fidelity


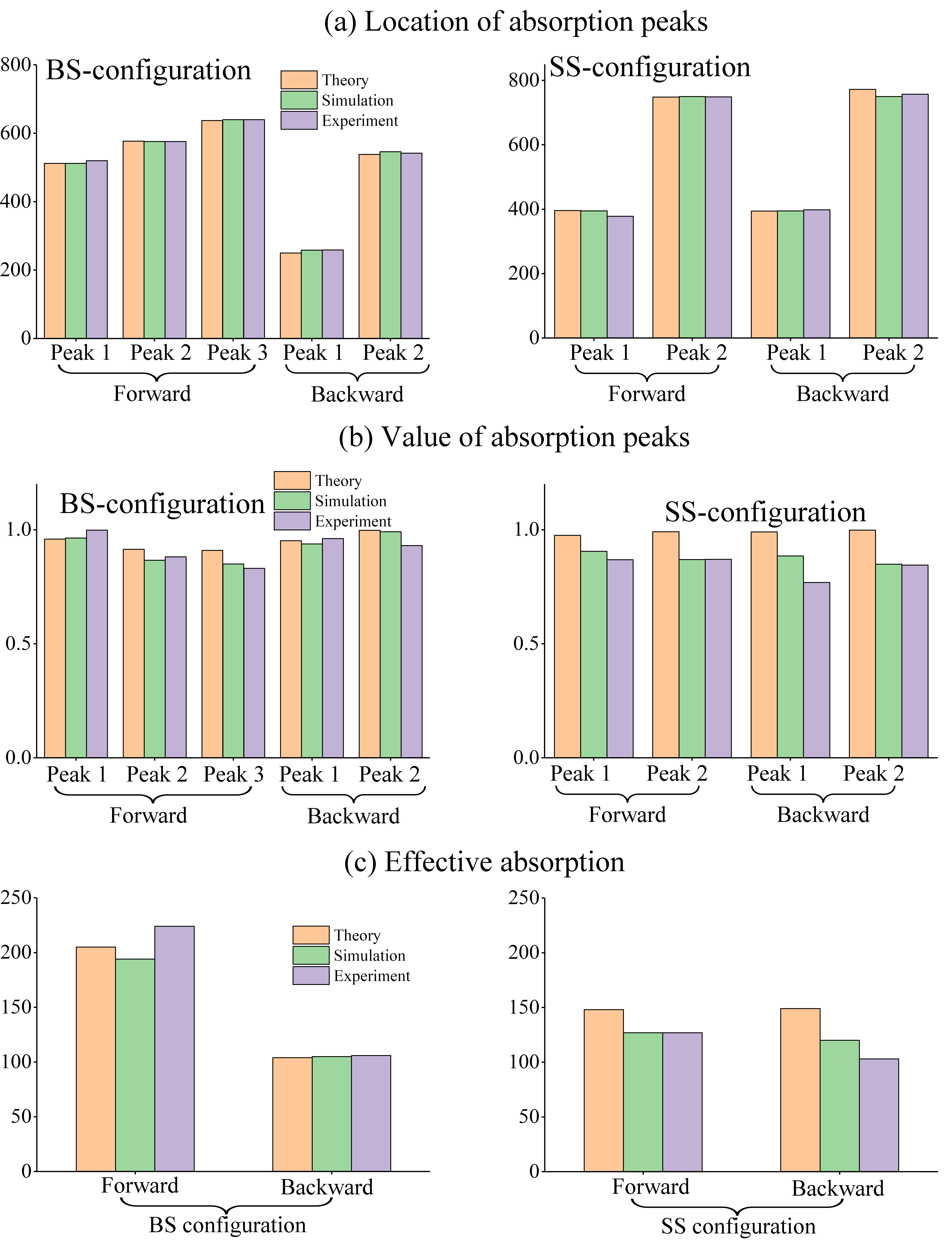


**Figure S9.** Quantitative comparison of absorption performance across theory, simulation, and experiment. (a) Location of absorption peaks for both SS- and BS-configurations under forward and backward incidence. (b) Value of absorption peaks for both SS- and BS-configurations under forward and backward incidence. (c) Effective absorption (α ≥ 0.5) for both SS- and BS-configurations under forward and backward incidence.

## Section S11. Analysis of experimental deviations.

FDM printing introduces melted edge deformation at perforated pores and surface texture on plate panels. These features, highlighted in red boxes and arrows, alter the effective pore size and introduce local roughness. Since the acoustic dissipation primarily arises from viscous losses around the micro-perforations, such manufacturing defects directly impact absorption efficiency ^[S8, S9]^. The increased surface roughness enhances viscous loss and promotes higher local energy dissipation, potentially resulting in minor shifts in experimental absorption peaks.


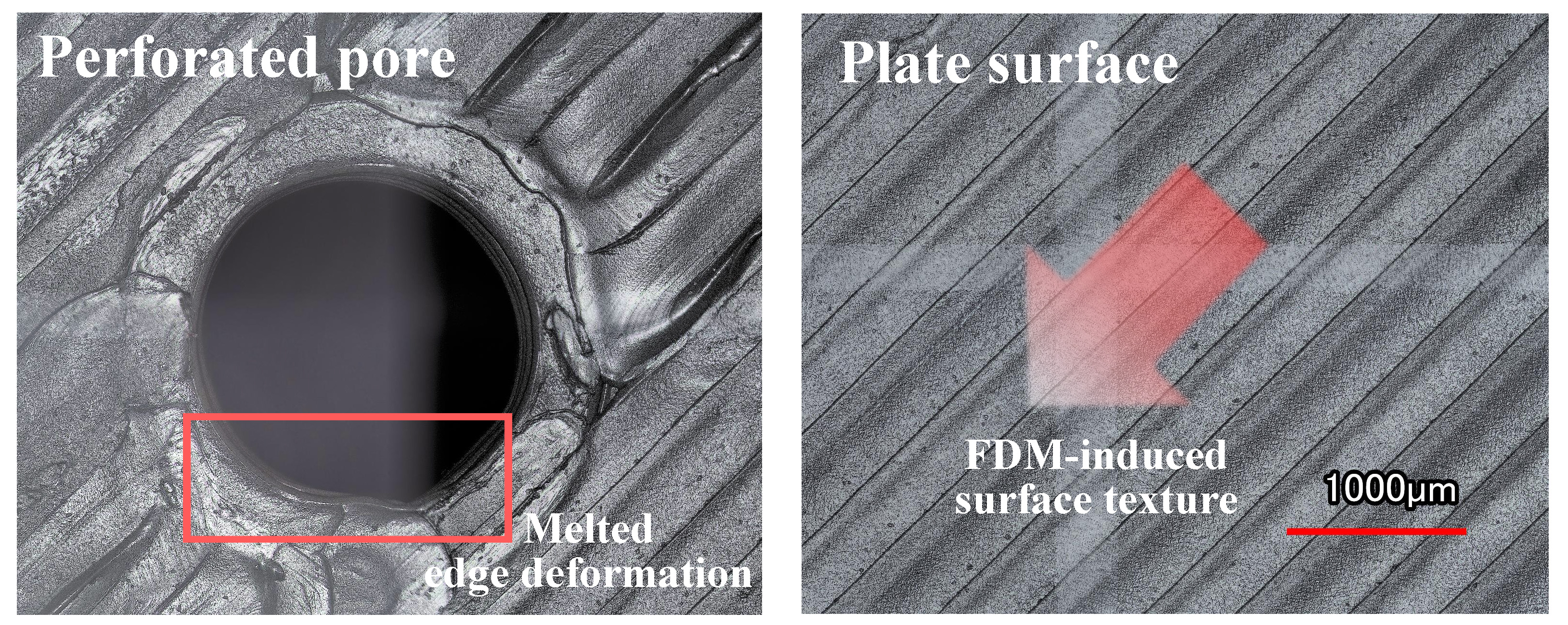


**Figure S11.** Representative fabrication-induced defects captured using a non-contact interferometry system (Keyence VK-X1000 Series).

**Reference**

1. Z. Guo, Z. Li, K. Zeng, J. Ye, X. Lu, Z. Lei, Z. Wang, *Adv. Mater. Technol.* **2025**, 10, 2400934
2. V. [Romero-García,](https://xueshu.baidu.com/s?wd=author%3A%28Romero-Garc%C3%ADa%2C%20V.%29%20&tn=SE_baiduxueshu_c1gjeupa&ie=utf-8&sc_f_para=sc_hilight%3Dperson) G. [Theocharis](https://xueshu.baidu.com/s?wd=author%3A%28G%20Theocharis%29%20&tn=SE_baiduxueshu_c1gjeupa&ie=utf-8&sc_f_para=sc_hilight%3Dperson), O. [Richoux](https://xueshu.baidu.com/s?wd=author%3A%28O%20Richoux%29%20&tn=SE_baiduxueshu_c1gjeupa&ie=utf-8&sc_f_para=sc_hilight%3Dperson), [A. Merkel](https://www.nature.com/articles/srep19519#auth-A_-Merkel-Aff1), [V. Tournat](https://www.nature.com/articles/srep19519#auth-V_-Tournat-Aff1), [V. Pagneux](https://www.nature.com/articles/srep19519#auth-V_-Pagneux-Aff1), *Sci. Rep* **2016**, 6(1), 19519.
3. C. Wang, W. R. Sweeney, A. D. Stone, L. Yang, *Science.* **2021**, 373, 1261–1265.
4. *ASTM E2611-17: Standard test method for normal incidence determination of porous material acoustical properties based on the transfer matrix method.*
5. [Yuanzhou Zhu](javascript:;), [Houyou Long](javascript:;), [Chen Liu](javascript:;), [Haixiao Zhang](javascript:;), [Ying Cheng](javascript:;), [Xiaojun Liu](javascript:;), *Appl. Phys. Lett* **2022**, 120, 141701.
6. A. Crivoi, L. Du, Z. Fan, *Appl. Acoust* **2023**, 205, 109263.
7. X. Wang, L. Du, Z. Liu, M. Li, Y. Weng, Z. Liu, Y. Tay, Z. Fan, T. Wong, M. Tan, *Virtual. Phys. Prototy* **2024**, 19:1, e2399787.
8. J. Boulvert, J. Costa-Baptista, T. Cavalieri, M. Perna, E. R. Fotsing, V. Romero-García, G. Gabard, A. Ross, J. Mardjono, J.-P. Groby, *Appl. Acoust* **2020**, 164, 107244.
9. X. Li, X. Yu, and W. Zhai, *Adv. Mater* **2021**, 2104552.
